# Zonisamide promotes survival of human‐induced pluripotent stem cell‐derived dopaminergic neurons in the striatum of female rats

**DOI:** 10.1002/jnr.24668

**Published:** 2020-06-07

**Authors:** Yoshifumi Miyawaki, Bumpei Samata, Tetsuhiro Kikuchi, Kaneyasu Nishimura, Jun Takahashi

**Affiliations:** ^1^ Department of Clinical Application Center for iPS Cell Research and Application Kyoto University Kyoto Japan; ^2^ Present address: Division of Integrated Pharmaceutical Sciences Kyoto Pharmaceutical University Kyoto Japan

**Keywords:** dopaminergic neuron, induced pluripotent stem cell, Parkinson's disease, RRID:AB_2169021, RRID:AB_2190153, RRID:AB_2201528, RRID:AB_2244867, RRID:AB_2294104, RRID:AB_2340866, RRID:AB_2534074, RRID:AB_2534102, RRID:AB_2534105, RRID:AB_2535795, RRID:AB_2535864, RRID:AB_2556542, RRID:AB_2556546, RRID:AB_2556547, RRID:AB_2716768, RRID:AB_2801320, RRID:AB_390204, RRID:AB_442102, RRID:AB_477010, RRID:AB_90755, RRID:AB_94090, RRID:CVCL_RL23, RRID:RGD_1547866, RRID:RGD_9685748, RRID:SCR_001456, RRID:SCR_002798, RRID:SCR_010972, RRID:SCR_016264, RRID:SCR_017202, RRID:SCR_017205, RRID:SCR_017206, transplantation, Zonisamide

## Abstract

The transplantation of dopaminergic (DA) progenitors derived from pluripotent stem cells improves the behavior of Parkinson's disease model animals. However, the survival of DA progenitors is low, and the final yield of DA neurons is only approximately 0.3%–2% the number of transplanted cells. Zonisamide (ZNS) increases the number of survived DA neurons upon the transplantation of mouse‐induced pluripotent stem (iPS) cell‐derived DA progenitors in the rat striatum. In this study, we induced DA progenitors from human iPS cells and transplanted them into the striatum of female rats with daily administration of ZNS. The number of survived DA neurons was evaluated 1 and 4 months after transplantation by immunohistochemistry, which revealed that the number of survived DA neurons was significantly increased with the administration of ZNS. To assess the mechanism of action of ZNS, we performed a gene expression analysis to compare the gene expression profiles in striatum treated with or without ZNS. The analysis revealed that the expression of SLIT‐and NTRK‐like protein 6 (SLITRK6) was upregulated in rat striatum treated with ZNS. In conclusion, ZNS promotes the survival of DA neurons after the transplantation of human‐iPS cell‐derived DA progenitors in the rat striatum. SLITRK6 is suggested to be involved in this supportive effect of ZNS by modulating the environment of the host brain.

## Graphical abstract

We examined the effects of zonisamide on transplanted human dopaminergic neurons in the rat striatum and found that zonisamide facilitates the survival of dopamine neurons, an effect that may be related to an increase in the expression of SLIT‐and NTRK‐like protein 6 in rat striatum.

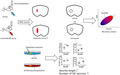

**Significance**

Cell transplantation therapy is expected to ameliorate the motor symptoms of Parkinson's disease. The survival of transplanted cells, however, is low, and the final yield of dopaminergic (DA) neurons is only about 0.3%–2% the number of the transplanted cells. This low yield demands a large number of cells and potentially increases the safety risks of the cell therapy. Here we show that Zonisamide promotes the survival of transplanted human‐induced pluripotent stem cell‐derived DA neurons in rat brains. Further observation suggested that its mode of action was associated with the increased expression of SLIT‐and NTRK‐like protein 6 in the host striatum.

## 1 INTRODUCTION

Parkinson's disease (PD) is a progressive neurodegenerative disease characterized by a loss of dopaminergic (DA) neurons in the substantia nigra. Major motor symptoms include tremors, muscular rigidity, akinesia, and postural reflex disturbance. Standard treatments for PD patients are drug administration and deep brain stimulation, but these treatments just relieve neurological symptoms and do not prevent the progressive degeneration of DA neurons.

As a more radical treatment, cell replacement of the lost DA neurons is being considered. The transplantation of fetal mesencephalic tissues has been performed since the 1980s and shows long‐term improvement of the PD symptoms (Kefalopoulou et al., 2014; Li et al., 2016). However, fetal mesencephalic tissues are difficult to acquire, limiting the number of patients who can benefit from this therapy. Pluripotent stem cells (PSCs) are easier to access, which is why they are expected to become another cell source for the transplantation (Barker, Drouin‐Ouellet, & Parmar, 2015). Another concern is that only a small fraction of grafted cells survive after transplantation, and the final yield of DA neuron is only about 0.3%–2% the number of the transplanted cells (Doi et al., 2014; Kikuchi et al., 2017). This result has important implications on the therapeutic benefit, because clinical trials using fetal mesencephalic tissues reported that the improvement of the clinical symptoms was related to the number of survived DA neurons in the striatum (Hagell & Brundin, 2001).

Zonisamide (ZNS) blocks calcium and sodium channels and is clinically used as an anticonvulsant. It also protects DA neurons from insults by neurotoxins such as rotenone and 1‐Methyl‐4‐phenyl‐1,2,3,6‐tetrahydropyridine and 6‐hydroxy dopamine (Asanuma et al., 2010; Choudhury et al., 2010; Condello et al., 2013; Czapiński, Blaszczyk, & Czuczwar, 2005) and is used as a symptomatic treatment for PD patients (Uemura, Asano, Hikawa, Yamakado, & Takahashi, 2017). ZNS has been reported not only to have these direct effects on DA neurons, but also to have indirect effects that promote the production of neurotrophic factors by astrocytes (Asanuma et al., 2010). Although previous reports showed neuroprotective effects of ZNS *in vivo* (Asanuma et al., 2010; Choudhury et al., 2010, 2012) and *in vitro* (Kawajiri, Machida, Saiki, Sato, & Hattori, 2010), the mechanism of action remains elusive.

A previous study reported that the intraperitoneal administration of ZNS increased the number of survived mouse‐induced pluripotent stem (iPS) cell‐derived DA neurons in the rat striatum 1 month after transplantation (Yoshikawa, Samata, Ogura, Miyamoto, & Takahashi, 2013). However, it is not known if ZNS exerts the same effect on human DA neurons. To investigate the effect of ZNS on human DA neurons and its mechanism, we induced DA progenitors from human iPS cells and grafted them into the rat striatum accompanied by ZNS administration. Histological analyses revealed that ZNS increased the number of survived DA neurons at 1 and 4 months post transplantation. In addition, a microarray analysis and a co‐culture experiment suggested that SLIT‐and NTRK‐like protein 6 (SLITRK6) plays a role in this effect.

## 2 MATERIALS AND METHODS

### 2.1 Human iPS cell culture

This study was approved by the ethics committee of Kyoto University, Kyoto, Japan. Human iPS cell line 1039A1 (XY, passages 15–25, RRID:CVCL_RL23) was maintained on E8 fragments of human laminin 511 (LM511) with Stem Fit AK02N (Cat# RCAK02N, Ajinomoto, Tokyo, Japan). When these cells were passaged, they were treated with TrypLE select (Cat# 12604013, Invitrogen, Carlsbad, CA, USA) and replated at a density of 3 × 10^4^ cells per well in a six‐well plate with Stem Fit AK02N medium containing 30 μM Y‐27632 (Cat# 030‐24026, Wako, Osaka, Japan).

### 2.2 Induction of DA progenitors from human iPS cells

The induction of DA progenitors from human iPS cells was performed as described previously (Doi et al., 2014). Briefly, when we started the neural induction, human iPS cells were dissociated into single cells with TrypLE select and replated on LM511‐coated six‐well plates at a density of 4 × 10^5^ cells with Stem Fit AK02N medium supplemented with 30 μM Y‐27632. After 4 days of culture in the maintenance medium, the medium was changed to differentiation medium containing Glasgow's MEM (GMEM, Cat# 11710‐035, Invitrogen) supplemented with 8% knockout serum replacement (KSR, Cat# 12618013, Invitrogen), 0.1 mM MEM non‐essential amino acids (Cat# 11140035, Invitrogen), 1 mM sodium pyruvate (Cat# 113‐24‐6, Sigma‐Aldrich, St. Louis, MO, USA), and 5 μM 2‐mercaptoethanol (Cat# 135‐14352, Wako). The day of the change was counted as differentiation Day 0. Additionally, 500 nM/A83‐01 (Cat# 035‐24113, Wako) and 100 nM LDN193189 (Cat# 1062368‐24‐4, Stemgent, Lexington, MA, USA) were added until Day 7 and Day 12, respectively. We also added 100 ng/ml of FGF8 (Cat# 069‐04373, Wako) and 2 μM purmorphamine (Cat# 483367‐10‐8, Wako) from Day 1 to Day 7 and 3 μM CHIR99021 (Cat# 04‐0004‐10, Stemgent) from Day 3 to Day 12. After cell sorting at Day 12, the sorted cells were replated in low cell adhesion 96‐well plates (Lipidure‐Coat Plate A‐96U, NOF, Tokyo, Japan) at a density of 2 × 10^4^ cells per well and cultured as neurospheres in neural differentiation medium containing Neurobasal Medium (Cat# 21103049, Gibco, Waltham, MA, USA) supplemented with B27 supplement (Cat# 17504044, Invitrogen), 100 units/ml and 100 μg/ml of penicillin/streptomycin (Cat# 15140122, Invitrogen), 2 mM l‐glutamine (Cat# 25030081, Invitrogen), 10 ng/ml of glial cell‐derived neurotrophic factor (GDNF, Cat# 074–06264, Wako), 200 nM of ascorbic acid (Cat# 50‐81‐7, Wako), 20 ng/ml of brain‐derived neurotrophic factor (BDNF, Cat# 218441‐99‐7, Wako), and 400 µM of dibutyryl cyclic adenosine monophosphate (dbcAMP, Cat# 16980‐89‐5, Sigma‐Aldrich). At Day 28, the neurospheres were used for the transplantation or *in vitro* analysis. For the co‐culture experiments, cells sorted at Day 12 were cultured in attachment condition with neural differentiation medium, and attached cells at Day 28 were dissociated and used.

### 2.3 Fluorescence‐activated cell sorting

To perform cell sorting, the cells were dissociated and stained with anti‐CORIN antibody (1:200) for 30 min and then Alexa 488‐conjugated donkey anti‐mouse IgG antibody for 30 min at Day 12 of the culture. The staining was performed at 4°C. To separate living cells from dead cells, the cells were also stained with 7‐amino‐actinomycin D (Cat# 559925, BD Biosciences, San Jose, CA, USA). Fluorescence‐activated cell sorting (FACS) analysis was performed using FACS Aria II (BD Biosciences), and the data were analyzed using FACSDiva software (BD Biosciences, RRID:SCR_001456). The sorted cells were replated in low adhesion U‐shaped 96‐well plates with neural differentiation medium containing 30 µM Y‐27632.

### 2.4 Cell transplantation

All animals were cared for and handled according to the Guidelines for Animal Experiments of Kyoto University. Cell transplantation was performed as described in our previous publication (Doi et al., 2014). F344‐*Il2rg^em2Kyo^* X‐SCID rats (NBRP‐Rat No.0586, Kyoto University, Japan, RRID:RGD_9685748; Mashimo et al., 2010) were used for the transplantation experiments. In this study, we used female animals because we previously showed that xenograft transplants did not cause an immune response in female X‐SCID rats (Samata et al., 2015). At Day 28, neurospheres were collected in 0.2‐mL microtubes, washed by saline and centrifuged. The neurospheres (5.0 × 10^5^ cells in 2 µl of saline per animal) were injected stereotactically through a 22G needle into the striatum of rats (from the bregma: A, +1.0 mm; L, ±2.5 mm; V, −5.0 mm, and 4.0 mm; TB, 0 mm). The rats were intraperitoneally injected with ZNS (30 or 60 mg/kg; a gift from Sumitomo Dainippon Pharma Co., Ltd.) or saline every day starting 2 days before the transplantation until the day of euthanization. One or 4 months after the transplantation, the rats were euthanized with pentobarbital (Cat# 11441, Kyoritsu Seiyaku, Tokyo, Japan) administration and perfused with 4% paraformaldehyde (Cat# 30525‐89‐4, Wako). Brains were post fixed for 1 day with 4% paraformaldehyde and then transferred to 15% sucrose solution for one more day and then 30% sucrose solution until the day of sectioning at 4°C. The brains were sliced with a cryostat (CM‐1850, Leica Biosystems, Wetzlar, Germany) at 30 µm thickness. A total of 60 rats were used in this study.

### 2.5 Immunofluorescence studies

For *in vitro* studies, spheres and attached cells were fixed with 4% paraformaldehyde for 10 min. The spheres were sliced with a cryostat at 20 µm thickness and mounted on glass slides. The fixed cells were incubated with phosphate‐buffered saline (PBS, Cat# 166‐23555, Wako) containing 2% Triton X‐100 (Cat# 9002‐93‐1, Sigma‐Aldrich) for 30 min and blocked with PBS containing 4% BlockAce (Cat# UKB80, Megmilk Snow Brand, Tokyo, Japan) at room temperature for 10 min. The primary antibody reaction was performed overnight at 4°C. The antibodies used are listed in Supporting Information Table S1. After washing with PBS twice, the samples were incubated with PBS containing 4% BlockAce and Alexa Fluor‐conjugated secondary antibodies at room temperature for 1 hr. After washing with PBS twice, the cells were incubated with PBS containing 4′,6‐diamidino‐2‐phenylindole (DAPI, Cat# D1306, Dako) for nuclear staining and mounted on glass slides using Mowiol 4‐88 (Cat# 81381, Sigma‐Aldrich). The cells were visualized with a confocal laser microscope (Fluoview FV1200 D; Olympus, Tokyo, Japan, RRID:SCR_016264) or a fluorescence microscope (BZ X‐710; Keyence, Osaka, Japan, RRID:SCR_017202). The number of TH^+^, NURR1^+^, FOXA2^+^, and KI67^+^ cells was counted using 30‐µm brain sections. One slice out of eight continuous sections was used for the cell count, and at least two sections per animal were counted. For human nuclei and SLITRK6, at least 300 cells in randomly selected 20× fields were counted.

### 2.6 Microarray analysis

For microarray analysis, female F344 rats (CLEA Japan, Inc., Japan, RRID:RGD_1547866) were intraperitoneally injected with 60 mg kg^−1^ day^−1^ of ZNS (*n* = 4) or saline (vehicle control, *n* = 2) for seven successive days. Then the rats were euthanized, total RNA was extracted from the striatum using an RNeasy Plus Mini Kit (Qiagen, Valencia, CA, USA), and 100 ng of total RNA was used for the analysis. Samples were hybridized to GeneChip Rat Gene 1.0 ST arrays (Affymetrix, Santa Clara, CA, USA) according to the manufacturer's protocol. Arrays were scanned using the Microarray Scanner System (Agilent Technologies, Santa Clara, CA, USA, RRID:SCR_017206). The data were analyzed using GeneSpring GX13.1.1 software (Agilent Technologies, RRID:SCR_010972). The expression signals of the probe sets were calculated using RMA16. The microarray data are available from the Gene Expression Omnibus (GEO database, http://www.ncbi.nlm.nih.gov/geo/) with the accession number GSE112624.

### 2.7 Quantitative RT‐PCR

Total RNA was extracted using an RNeasy Plus Mini Kit. The samples were reverse transcribed using the SuperScript III First‐Strand Synthesis System (Cat# 18080051, Invitrogen). Quantitative PCR reactions were carried out with a Power SYBR (Cat# 4368577, Applied Biosystems, Foster City, CA, USA) according to the manufacturer's instructions. The expression level of each gene was normalized to that of GAPDH using the ΔΔCt method. The primers used are shown in Supporting information Table S2.

### 2.8 Generation of HEK293T cells forcibly expressing SLITRK6 (SLITRK6‐HEK293T cells)

A pPV‐EF1a‐SLITRK6iP‐A vector was transfected into HEK293T cells by Lipofectamine 2000 reagent (Cat# 12566014, Thermo Fisher Scientific, Waltham, MA, USA), and the cells were maintained in DMEM/F‐12 Ham with l‐Glutamine and Phenol Red (Cat# 12‐719Q, Wako) supplemented with 10% fetal bovine serum (Cat# 11550356, Sigma‐Aldrich). After 24 hr, 2.5 μg/ml of puromycin (Cat# 58‐58‐2, InvivoGen, San Diego, CA, USA) was added to the culture medium to select drug‐resistant cells.

### 2.9 Co‐culture of SLITRK6‐HEK293T cells and iPS cell‐derived DA progenitors

Three days prior to co‐culture, SLITRK6‐HEK293T or HEK293T cells were dissociated into single cells with Accumax (Cat# AM105, Innovative Cell Technologies, San Diego, CA, USA) and replated on poly‐L‐lysine‐coated eight‐well slide chamber plates at a density of 4.5 × 10^5^ cells with Neurobasal Medium supplemented with B27 supplement and 2 mM l‐glutamine. When we started the co‐culture, attached Day‐28 DA progenitors were dissociated into single cells with Accumax and replated on SLITRK6‐HEK293T or HEK293T cultivated plates at a density of 1.5 × 10^5^ cells. At Day 4 of the co‐culture, the samples were fixed with 4% paraformaldehyde.

### 2.10 Quantitation of fiber length

After immunostaining, TH^+^ fibers were measured by a BZ Analyzer II (Keyence, RRID:SCR_017205). Ten fibers per sample were randomly selected and measured, and the average value was considered the value for that sample.

### 2.11 Oxidative stress test

At Day 28 of the culture, neurosphere media were changed to Neurobasal Medium minus phenol red (Cat# 12348‐017, Gibco) supplemented with B‐27 Supplement Minus AO (Cat# 10889038, Gibco) and 2 mM l‐glutamine. ZNS was added at three different concentrations: 0.05, 0.5, and 5 µM. After 1 day of ZNS exposure, the cells were incubated with 1 mM H_2_O_2_ for 12 hr. The viability of the cells was examined with an LDH Cytotoxicity Detection Kit (Cat# MK401, Takara Bio, Shiga, Japan) according to the manufacturer's protocol.

### 2.12 Maturation test

At Day 28 of the culture, neurosphere media were changed to Neurobasal Medium supplemented with B27 supplement and 2 mM l‐glutamine. The cells were incubated with 10 or 100 µM ZNS for 14 days. At Day 42, the neurospheres were fixed with 4% paraformaldehyde and subjected to immunofluorescence staining.

### 2.13 Statistical analysis

Statistical analyses were performed using a commercially available software package (GraphPad Prism, GraphPad Software, San Diego, CA, USA, RRID:SCR_002798). Differences were considered statistically significant when *p* < 0.05. Data from the analysis of brain slices 1 month after transplantation and neurospheres were analyzed by one‐way ANOVA with the Bonferroni multiple comparison test (Figures 2b,d,e, 3b, and 4a,c). Data from the analysis of brain slices 4 months after transplantation, qPCR, and co‐culture experiments were compared using an unpaired *t* test (Figures 2g,2, 3, 5, 6, 8, 3d, 5b, 6c,2, 3, 5, 6, 8). Data are shown as the mean ± standard deviation. *n* represents the number of independent experiments.

## 3 RESULTS

### 3.1 Differentiation of human iPS cells to DA progenitors

**FIGURE 1:**
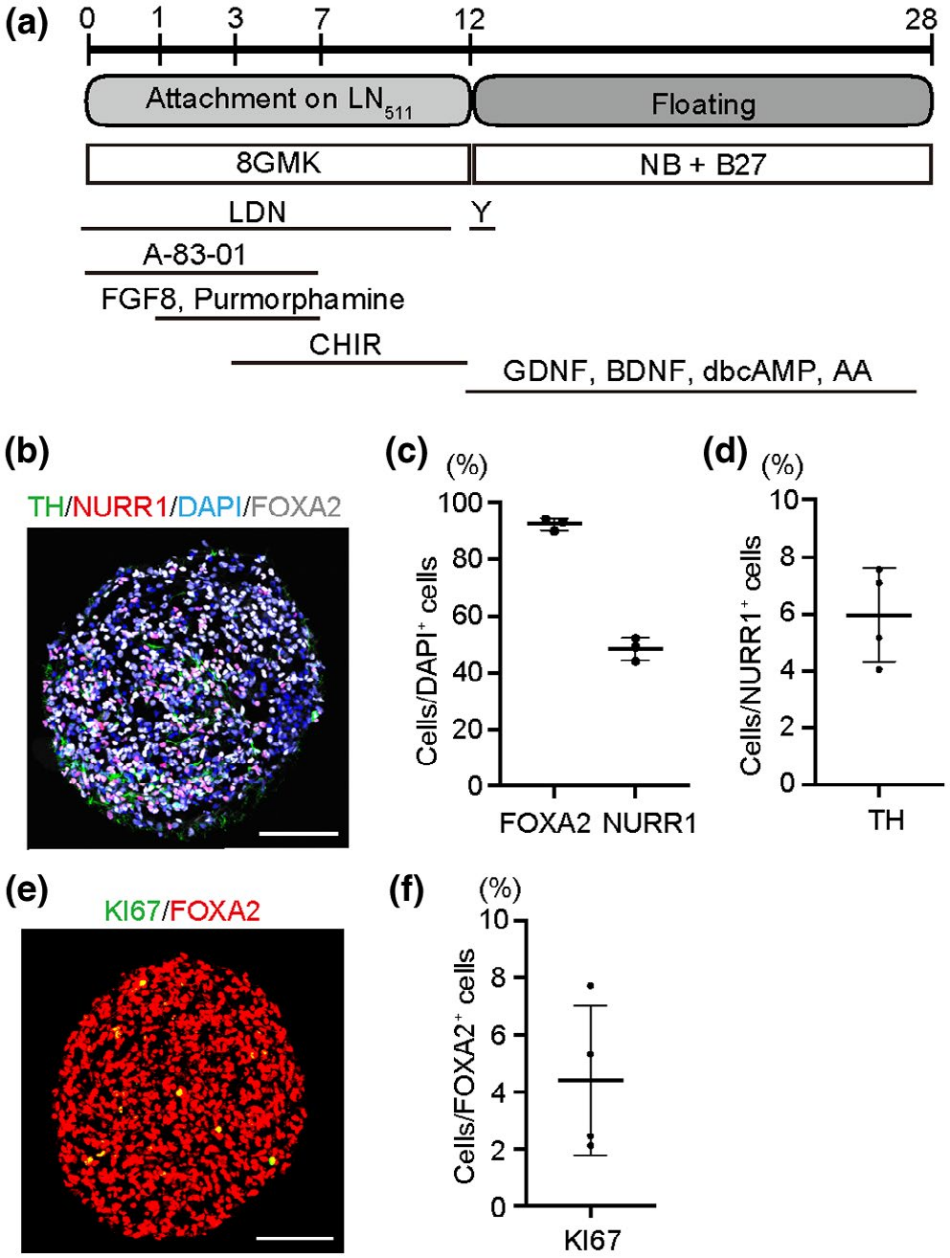
*In vitro* evaluation of differentiated dopaminergic (DA) progenitors. (a) Scheme of DA neuron differentiation from human iPS cells. LM511: E8 fragments of human laminin 511, 8GMK: GMEM supplemented with 8% knockout serum replacement, NB: Neurobasal Medium, LDN: LDN‐193189, Y: Y‐27632, CHIR: CHIR99021, GDNF: glial cell‐derived neurotrophic factor, BDNF: brain‐derived neurotrophic factor, dbcAMP: dibutyryl cyclic adenosine monophosphate, AA: ascorbic acid. (b) Immunofluorescence image of a neurosphere at Day 28 for TH (green), NURR1 (red), DAPI (blue), and FOXA2 (white). Scale bar = 100 µm. (c) Quantitation of FOXA2^+^ cells per total cells and NURR1^+^ cells per total cells (*n* = 3). (d) Quantitation of TH^+^ cells among NURR1^+^ cells (*n* = 4). (e) Immunofluorescence image of a neurosphere at Day 28 for KI67 (green) and FOXA2 (red). Scale bar = 100 µm. (f) Quantitation of KI67^+^ cells among FOXA2^+^ cells (*n* = 4)

We induced DA progenitors from human iPS cells with the dual SMAD inhibition‐based method (Chambers et al., 2009) (Figure 1a). On Day 12, we sorted Corin (a marker for floor plate)^+^ cells to enrich DA progenitors (Doi et al., 2014). Immunostaining on Day 28 revealed that forkhead box protein A2 (FOXA2, a marker for ventral cells)^+^ and nuclear receptor related 1 protein (NURR1, a marker for early midbrain DA neurons)^+^ cells were 92.3 ± 2.1% and 48.3 ± 4.0% (*n* = 3), respectively (Figure 1b,c). With this method, most of the differentiated cells were LIM homeobox transcription factor 1 alpha (LMX1A, midbrain marker)^+^ and FOXA2^+^ DA neuronal progenitors (Doi et al., 2014). The percentage of tyrosine hydroxylase (TH, a marker for postmitotic DA neurons)^+^ cells among NURR1^+^ cells was 6.0 ± 1.6% (*n* = 4) in Day‐28 spheres (Figure 1d), while the percentage of KI67^+^ cells among FOXA2^+^ cells was 4.4 ± 2.6% (*n* = 4) (Figure 1e,f).

### 3.2 ZNS promoted survival of DA neurons 1 month after transplantation

**FIGURE 2:**
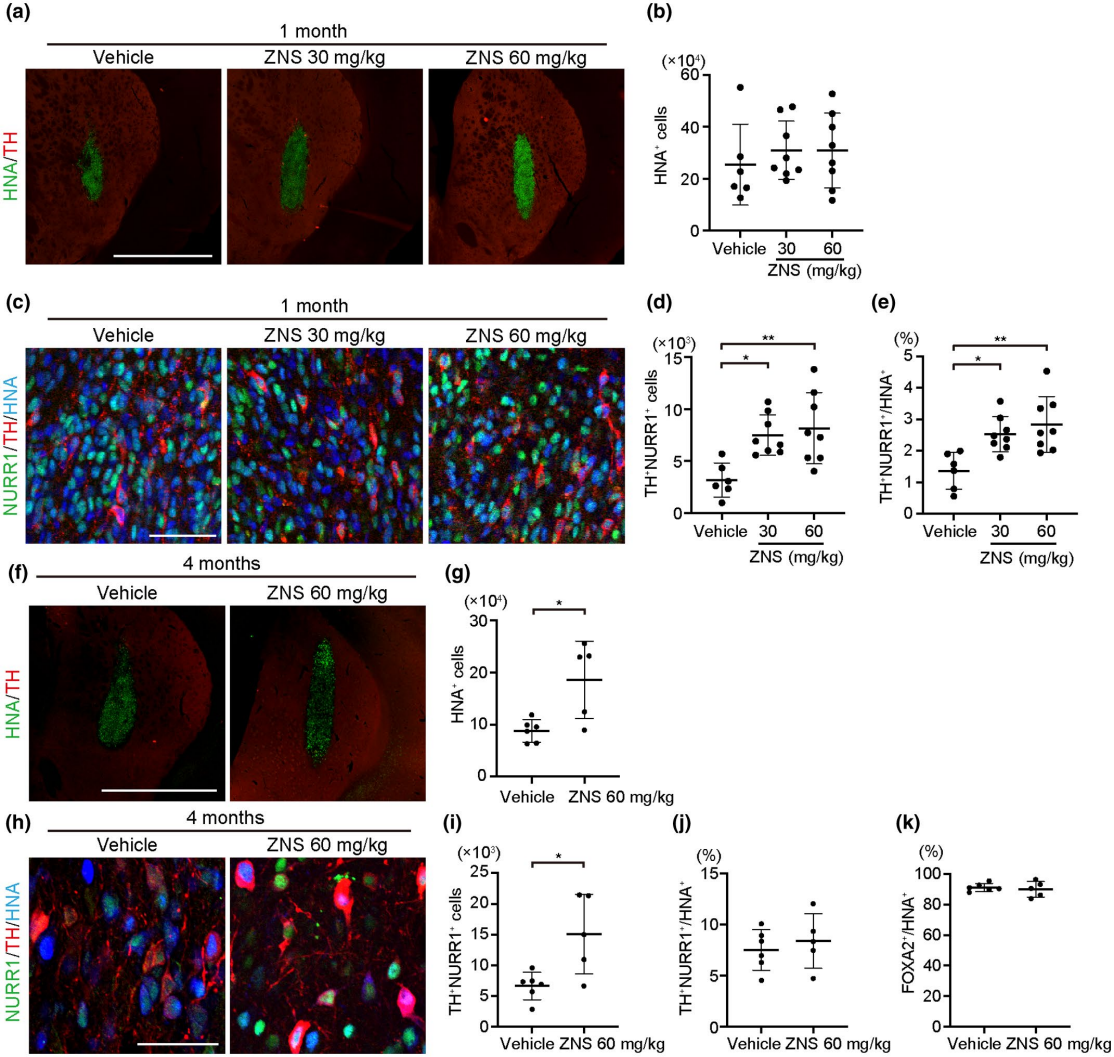
Zonisamide (ZNS) promotes survival of injected dopaminergic progenitors after transplantation into rat striatum. (a) Immunofluorescence images of grafted cells 1 month after transplantation for HNA (green) and TH (red). Scale bar = 2 mm. (b) Quantitation of HNA^+^ cells per graft 1 month after transplantation. Saline (vehicle control, *n* = 6), ZNS 30 mg/kg (*n* = 8), and ZNS 60 mg/kg (*n* = 8). (c) Immunofluorescence images of grafted cells 1 month after grafting for NURR1 (green), TH (red), and HNA (blue). Scale bar = 50 µm. (d) Quantitation of TH^+^NURR1^+^ cells per graft at 1 month after transplantation. Saline (*n* = 6), ZNS 30 mg/kg (*n* = 8), and ZNS 60 mg/kg (*n* = 8), *p < 0.05, **p < 0.01. (e) Quantitation of TH^+^NURR1^+^ cells among HNA^+^ cells in the graft 1 month after transplantation. Saline (*n* = 6), ZNS 30 mg/kg (*n* = 8), and ZNS 60 mg/kg (*n* = 8), *p < 0.05, **p < 0.01. (f) Immunofluorescence images of grafted cells 4 months after transplantation for HNA (green) and TH (red). Scale bar = 2 mm. (g) Quantitation of HNA^+^ cells per graft 4 months after transplantation. Saline (*n* = 6) and ZNS (*n* = 5), *p < 0.05. (h) Immunofluorescence images of grafted cells 4 months after transplantation for NURR1 (green), TH (red), and HNA (blue). Scale bar = 50 µm. (i) Quantitation of TH^+^NURR1^+^ cells per graft 4 months after transplantation. Saline (*n* = 6) and ZNS (*n* = 5), *p < 0.05. (j) Quantitation of TH^+^NURR1^+^ cells among HNA^+^ cells in the graft 4 months after transplantation. Saline (*n* = 6) and ZNS (*n* = 5). (k) Quantitation of FOXA2^+^ cells among HNA^+^ cells in the graft 4 months after transplantation. Saline (*n* = 6) and ZNS (*n* = 5)

We transplanted Day‐28 spheres into the rat striatum and administered saline (vehicle control) or ZNS (30 and 60 mg/kg). Immunohistological analyses at 1 month revealed that the number of human nuclear antigen (HNA)^+^ cells was 2.54 ± 1.56 × 10^5^, 3.10 ± 1.12 × 10^5^, and 3.08 ± 1.44 × 10^5^ cells for the vehicle (*n* = 6), ZNS 30 mg/kg (*n* = 8) and 60 mg/kg (*n* = 8) groups, respectively (ANOVA [*F*(2, 19) = 0.349, *p* = 0.710]; Figure 2a,b). To assess the number of mature DA neurons, we performed double‐labeled immunostaining for TH and NURR1 (Figure 2c). The number of TH^+^NURR1^+^ cells was significantly higher in the ZNS groups compared to the vehicle group (3.17 ± 1.64 × 10^3^, 7.50 ± 1.95 × 10^3^, and 8.16 ± 3.43 × 10^3^ cells for vehicle, ZNS 30 and 60 mg/kg, respectively, ANOVA [*F*(2, 19) = 7.474, *p* = 0.004]; *post hoc* Bonferroni multiple comparison test: vehicle versus. ZNS30, *p* = 0.016, vehicle versus ZNS60, *p* = 0.005; Figure 2d). Similarly, the percentage of TH^+^NURR1^+^ cells among HNA^+^ cells was significantly higher in the ZNS groups (1.37 ± 0.59%, 2.53 ± 0.56%, and 2.84 ± 0.89% for vehicle, ZNS 30 mg/kg, and 60 mg/kg, respectively, ANOVA [*F*(2, 19) = 8.052, *p* = 0.003]; *post hoc* Bonferroni multiple comparison test: vehicle versus. ZNS30, *p* = 0.019, vehicle versus ZNS60, *p* = 0.003; Figure 2e). Thus, ZNS administration increased the number of survived DA neurons in the rat striatum 1 month after transplantation.

### 3.3 ZNS promoted survival of DA neurons 4 months after transplantation

From the above results, we continued observation of the ZNS 60 mg/kg group and examined the survival of DA neurons with or without ZNS (60 mg/kg) 4 months after transplantation. The number of survived HNA^+^ cells was significantly higher in the ZNS 60 mg/kg (*n* = 5) group compared to the vehicle (*n* = 6) group (8.80 ± 2.16 × 10^4^ and 1.86 ± 0.74 × 10^5^ cells for vehicle and ZNS 60 mg/kg groups, respectively, *p* = 0.012; Figure 2f,g). Again, the number of TH^+^NURR1^+^ cells was significantly higher in the ZNS 60 mg/kg group (6.64 ± 2.24 × 10^3^ and 1.51 ± 0.65 × 10^4^ cells for vehicle and ZNS 60 mg/kg, respectively, *p* = 0.015; Figure 2h,i). However, there was no significant difference in the percentage of TH^+^NURR1^+^ cells among HNA^+^ cells between the two groups (7.52 ± 1.99% and 8.37 ± 2.67% for vehicle and ZNS 60 mg/kg, respectively, *p* = 0.557; Figure 2j). Additionally, most of the HNA^+^ cells were FOXA2^+^ (91.3 ± 2.6% and 90.1 ± 5.1% for vehicle and ZNS 60 mg/kg, respectively, *p* = 0.624; Figure 2k). Thus, at 4 months, ZNS administration promoted the survival of the grafted cells including DA neurons.

### 3.4 ZNS did not promote proliferation of DA progenitors

**FIGURE 3:**
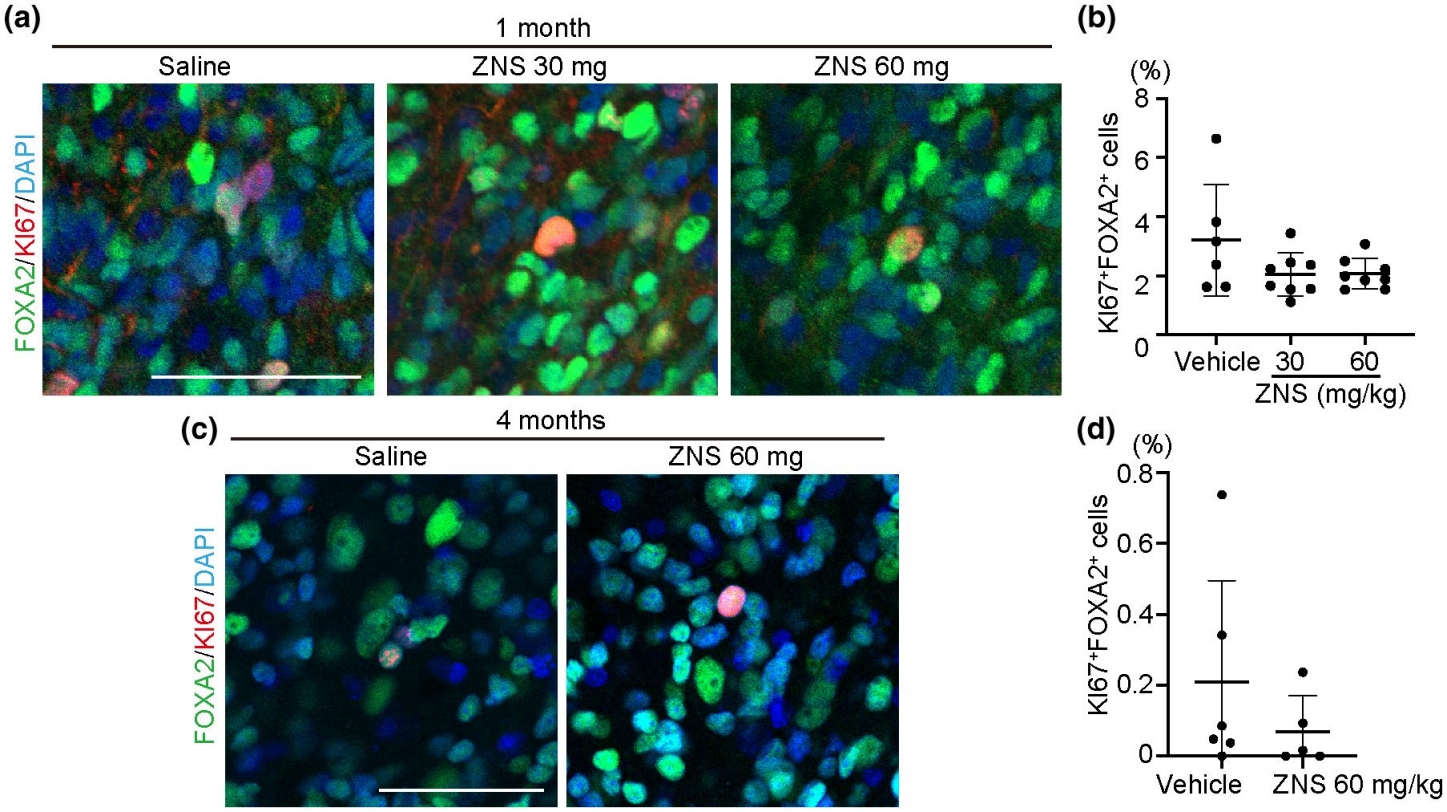
Zonisamide (ZNS) does not promote proliferation of DA progenitors. (a) Immunofluorescence images of grafted cells 1 month after transplantation for FOXA2 (green), KI67 (red), and DAPI (blue). Scale bar = 50 µm. (b) Quantitation of KI67^+^ cells among FOXA2^+^ cells 1 month after transplantation. Saline (vehicle control, *n* = 6), ZNS 30 mg/kg (*n* = 8), and ZNS 60 mg/kg (*n* = 8). (c) Immunofluorescence images of grafted cells 4 months after transplantation for FOXA2 (green), KI67 (red), and DAPI (blue). Scale bar = 50 µm. (d) Quantitation of KI67^+^ cells among FOXA2^+^ cells 4 months after transplantation. Saline (*n* = 6) and ZNS (*n* = 5)

We examined whether the increase of survived DA neurons was a result of promoted DA progenitor proliferation. We assessed cell proliferation by quantifying the percentage of KI67^+^ cells among FOXA2^+^ cells. At 1 month, there was no significant difference in proliferating midbrain DA progenitors among the vehicle and ZNS groups (3.21 ± 1.89%, 2.05 ± 0.73%, and 2.07 ± 0.52% for vehicle (*n* = 6), ZNS 30 mg/kg (*n* = 8) and 60 mg/kg (*n* = 8), respectively, ANOVA [*F*(2, 19) = 2.345, *p* = 0.123]; Figure 3a,b). At 4 months after transplantation, the proliferating DA progenitors were much fewer, and there was no significant difference between the groups (0.21 ± 0.29% and 0.07 ± 0.10% for vehicle (*n* = 6) and ZNS 60 mg/kg (*n* = 5), respectively, *p* = 0.332; Figure 3c,d). Therefore, we concluded that ZNS does not promote the proliferation of midbrain DA progenitors.

### 3.5 ZNS promotes the expression of *SLITRK6* in rat striatum

**FIGURE 4:**
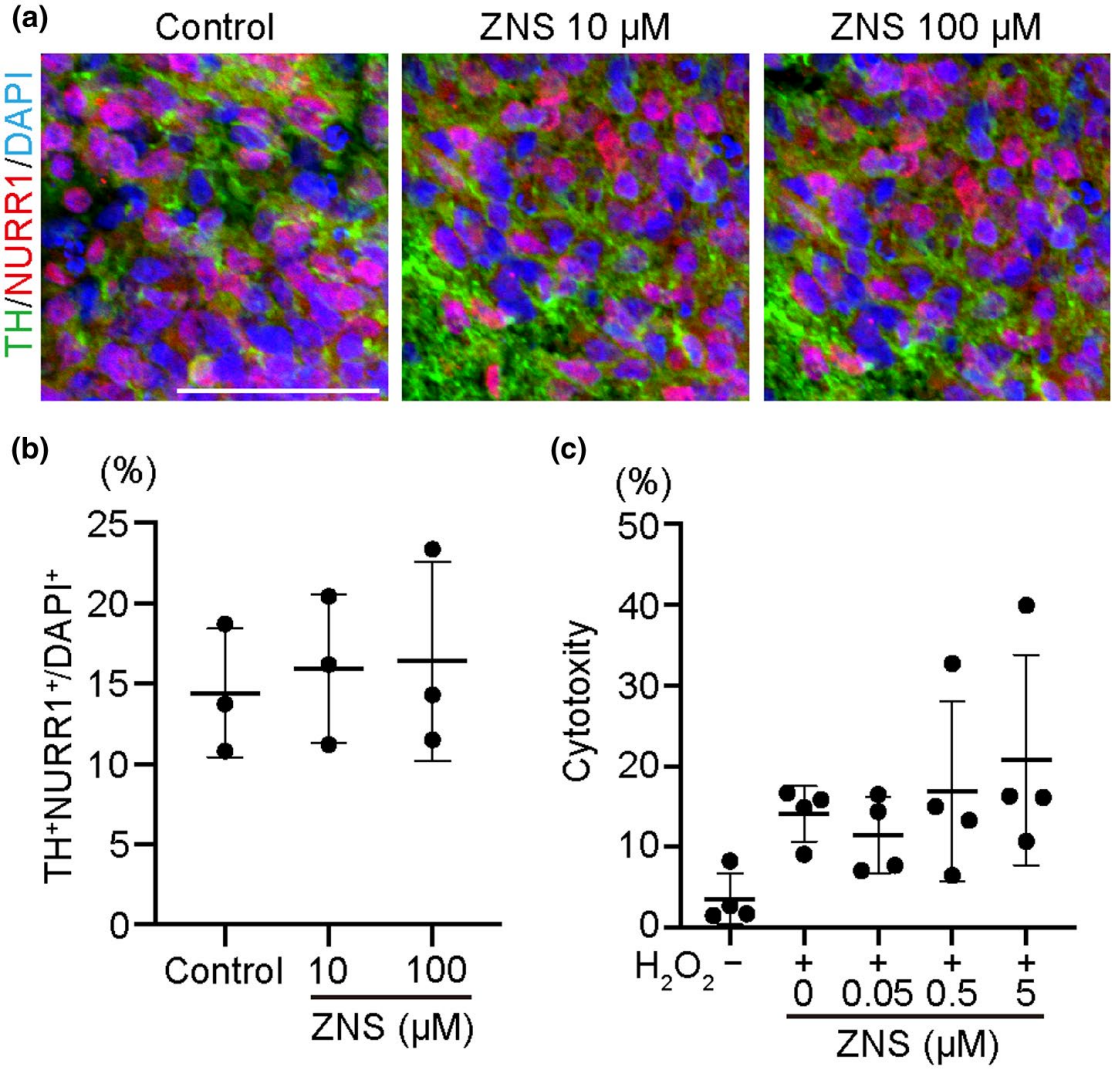
A Zonisamide (ZNS) direct effect was not observed by maturation or oxidative stress test. (a) Immunofluorescence images of sections of neurospheres at Day 42 (after 14 days exposure to ZNS). TH (green), FOXA2 (red), and DAPI (blue). Scale bar = 50 µm. (b) Quantitation of TH^+^NURR1^+^ cells of the 42‐day culture. Control (*n* = 3), ZNS 10 µM (*n* = 3) and ZNS 100 µM (*n* = 3). (c) Culture supernatants were collected and analyzed for LDH activity 12 hr after adding H_2_O_2_. H_2_O_2_− (*n* = 4), ZNS 0 µM (*n* = 4), ZNS 0.05 µM (*n* = 4), ZNS 0.5 µM (*n* = 4), and ZNS 5 µM (*n* = 4)

To determine the mechanism for how ZNS supports the survival of DA neurons, we first examined the direct effect of ZNS on DA progenitors. We cultured Day‐28 spheres with or without ZNS for another 14 days, but found no increase of TH^+^NURR1^+^ cells in the ZNS groups (ANOVA [*F*(2, 6) = 0.127, *p* = 0.883], Figure 4a,b). Then we examined if ZNS rescues DA progenitors from oxidative stress. We quantified cell death in Day‐28 spheres in the presence of H_2_O_2_ for 12 hr, but did not find any supportive effect of ZNS (ANOVA [*F*(4, 15) = 2.475, *p* = 0.089]; Figure 4c). These results suggested that any direct effect on DA neurons by ZNS is not likely the main mechanism that supports cell survival.

**FIGURE 5:**
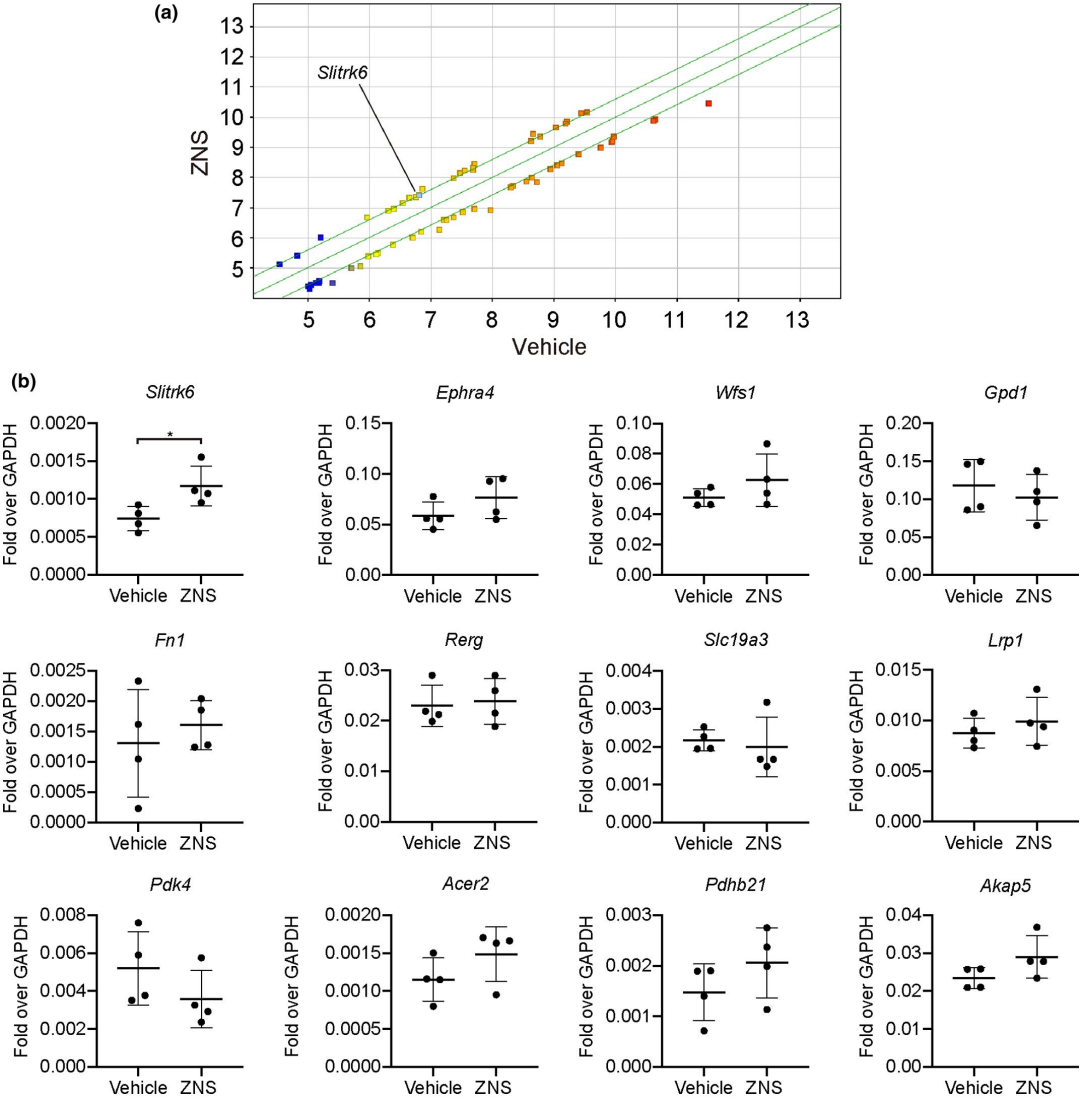
Gene expression analysis of rat striatum. (a) Scatter plot of the microarray analysis. Genes with fold change of more than 1.5 are shown. Light blue square indicates SLIT‐and NTRK‐like protein 6 (*SLITRK6*). (b) Quantitative PCR analysis of rat striatum for candidate genes after 7 days administration of Zonisamide (ZNS). Values are shown as fold over GAPDH. Vehicle (*n* = 4) and ZNS (*n* = 4), *p < 0.05

**FIGURE 6:**
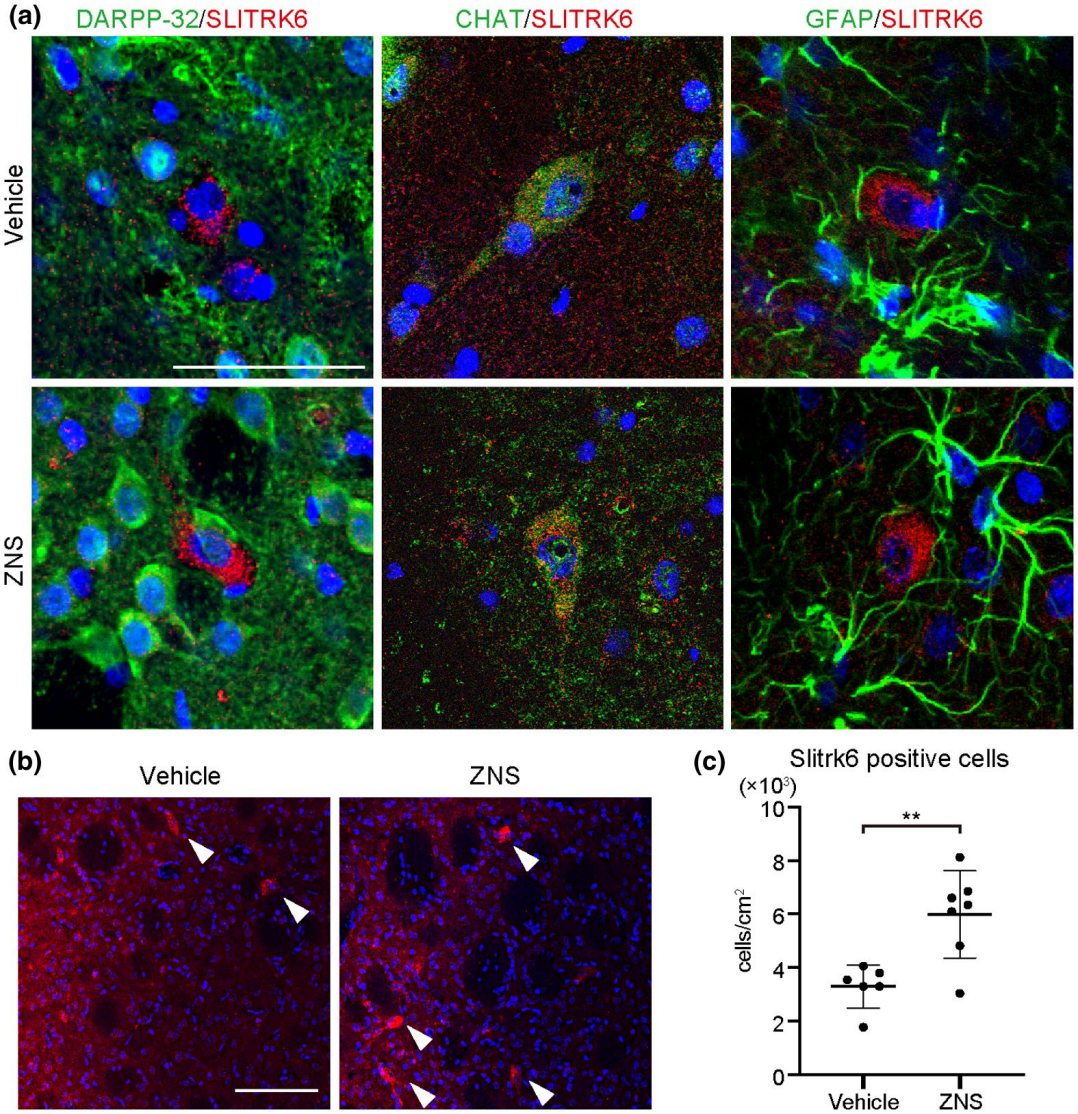
Zonisamide (ZNS) promotes expression of SLIT‐and NTRK‐like protein 6 (SLITRK6). (a) Immunofluorescence images of rat striatum 1 month after the administration of saline (vehicle control) or ZNS for DARPP‐32, CHAT, GFAP (green), SLITRK6 (red), and DAPI (blue). Scale bar = 50 µm. (b) Immunofluorescence images of rat striatum 1 month after the administration of saline or ZNS for SLITRK6 (red) and DAPI (blue). Scale bar = 100 µm. Arrowheads designate SLITRK6^+^ cells. (c) Quantitation of SLITRK6^+^ cells per cm^2^ 1 month after the administration of saline (*n* = 6) or ZNS (*n* = 7), **p < 0.01

Next, in order to assess the indirect effect of ZNS, we performed a microarray analysis of the intact rat striatum with or without ZNS administration. A comparison between these two groups disclosed 26 genes that were upregulated in the ZNS group more than 1.5 times compared to the vehicle group (Figure 5a). After combining synonymous genes using RefSeq and Gene Symbol, 13 genes remained as candidates. Among them, 12 genes were identified to encode membrane or secreted proteins. We examined the expression of the 12 genes by quantitative RT‐PCR and found that *SLITRK6* was significantly upregulated in the ZNS group (*n* = 4) compared to the vehicle group (*n* = 4) (*p* = 0.031; Figure 5b). To determine what kind of cells express SLITRK6, we performed an immunofluorescence study of the intact rat striatum with or without ZNS administration for one successive month. SLITRK6 was expressed in choline acetyltransferase (CHAT, a marker for cholinergic neurons)^+^ cells, but not in DARPP‐32 (a marker for striatal medium spiny neurons)^+^ or glial fibrillary acidic protein (GFAP, a marker for astrocytes)^+^ cells (Figure 6a). There was a significant difference in the number of SLITRK6^+^ cells in the striatum between the vehicle (*n* = 6) and the ZNS groups (*n* = 7) (3.30 ± 0.80 × 10^3^ and 5.98 ± 1.62 × 10^3^ cells/cm^2^ for vehicle and ZNS 60 mg/kg, respectively, *p* = 0.004; Figure 6b,c).

### 3.6 SLITRK6 supports survival of DA neurons *in vitro*

**FIGURE 7:**
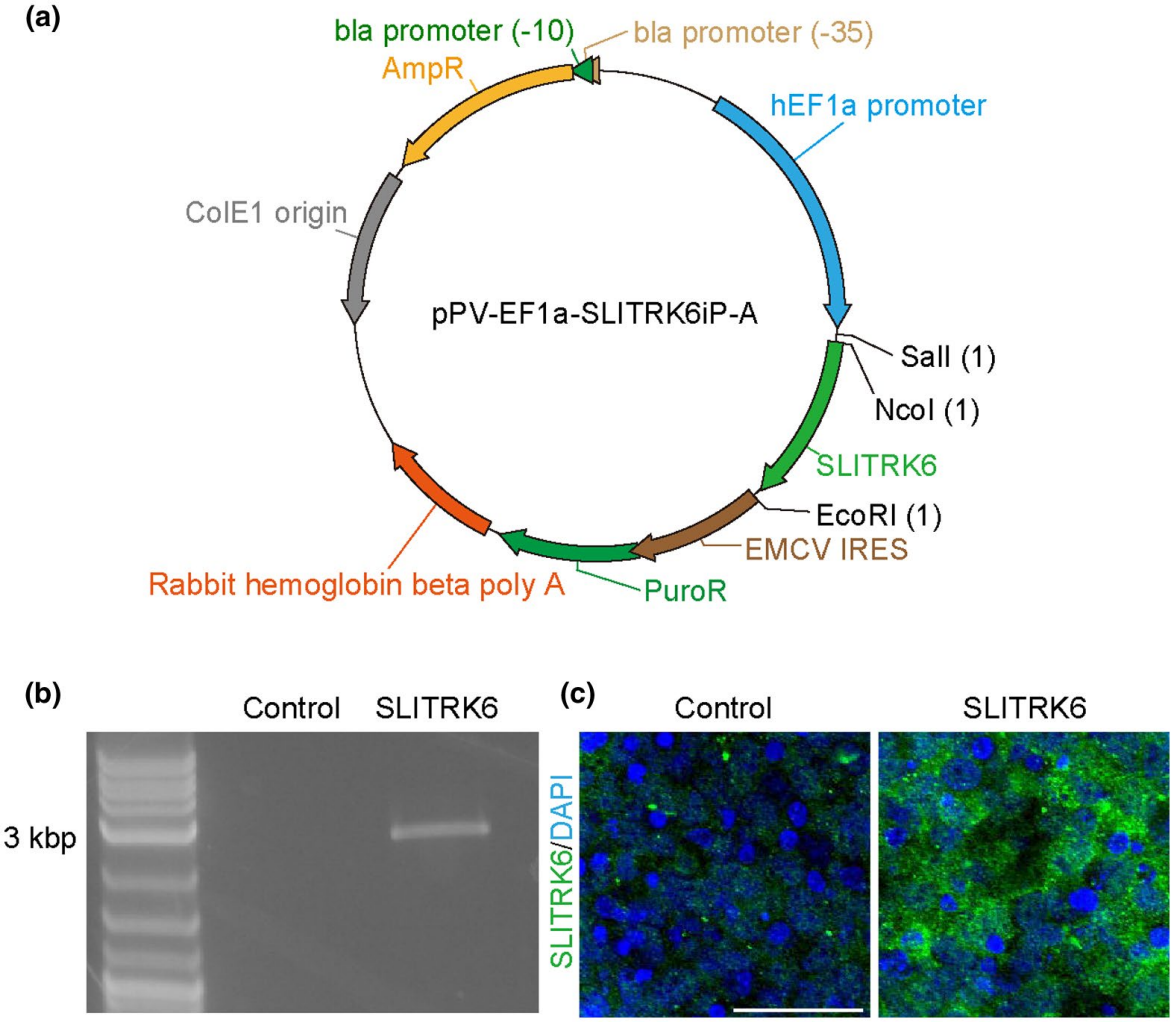
Establishment of SLIT‐and NTRK‐like protein 6 (SLITRK6)‐expressing HEK293T cell line. (a) Schematic drawing of the construct of the pPV‐EF1a‐SLITRK6iP‐A vector. (b) Reverse transcription PCR analysis for HEK293T and SLITRK6‐HEK293T cells. PCR product (2,561 bp) was observed in a SLITRK6‐HEK293T sample. (c) Immunofluorescence images of HEK293T and SLITRK6‐HEK293T cells. SLITRK6 (green) and DAPI (blue). Almost all SLITRK6‐HEK293T cells expressed SLITRK6. Scale bar = 50 µm

**FIGURE 8:**
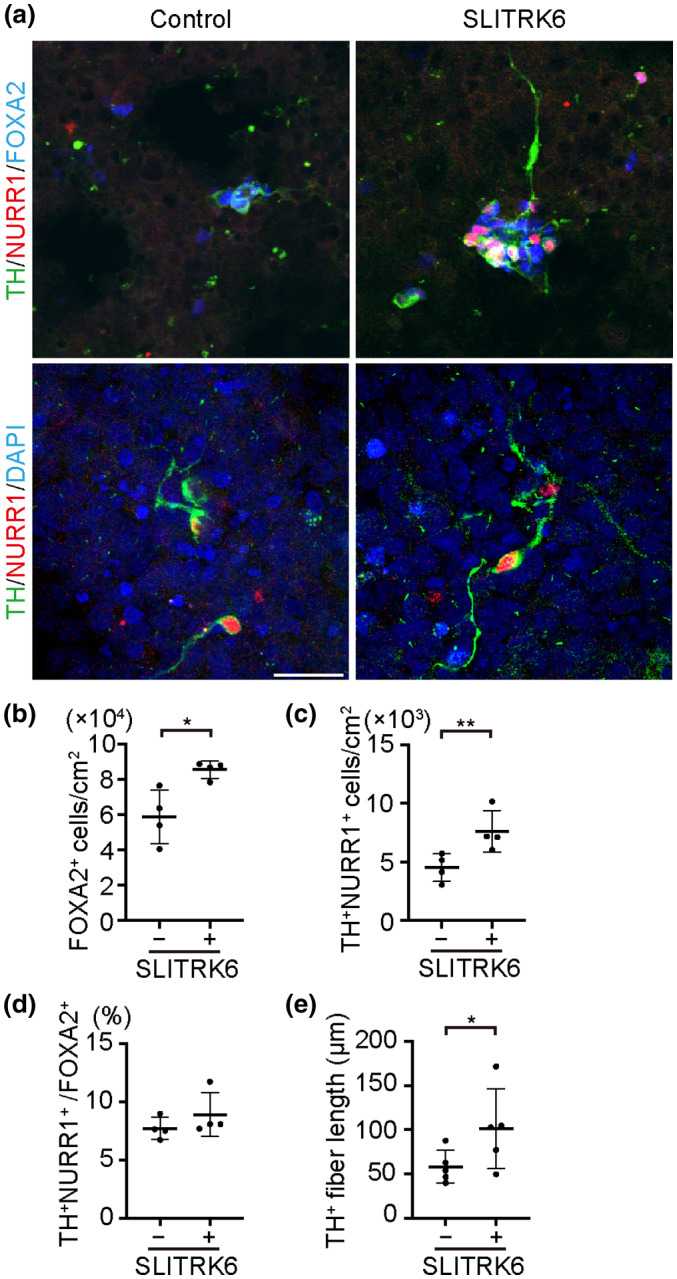
SLIT‐and NTRK‐like protein 6 (SLITRK6) promotes the survival and neurite extension of DA neurons. (a) Immunofluorescence images of samples after 4 days of co‐culture with HEK293T or SLITRK6‐HEK293T. TH (green), NURR1 (red), and DAPI (blue). Scale bar = 50 µm. (b) Quantitation of FOXA2^+^ cells per cm^2^ after co‐culture for 4 days. Saline (vehicle control, *n* = 4) and ZNS (*n* = 4), *p < 0.05. (c) Quantitation of TH^+^NURR1^+^ cells per cm^2^ after co‐culture for 4 days. Saline (*n* = 4) and Zonisamide (ZNS) (*n* = 4), **p < 0.01. (d) Quantitation of TH^+^NURR1^+^ cells per FOXA2^+^ cells after co‐culture for 4 days. Saline (*n* = 4) and ZNS (*n* = 4). (e) Quantitation of TH^+^ fiber length after co‐culture for 4 days. Saline (*n* = 5) and ZNS (*n* = 5), *p < 0.05

To assess the effects of SLITRK6 on DA neurons, we generated SLITRK6‐HEK293T cells (Figure 7). We dissociated Day‐28 spheres, plated them onto SLITRK6‐HEK293T cells and then performed an immunofluorescence study after 4 days (Figure 8a). The number of FOXA2^+^ cells was significantly higher in the SLITRK6 group (*n* = 4) compared to the control group (*n* = 4) (8.54 ± 0.48 × 10^4^ and 5.86 ± 1.52 × 10^4^ cells/cm^2^, respectively, *p* = 0.019; Figure 8b). Similarly, the number of TH^+^NURR1^+^ cells was significantly higher in the SLITRK6 group compared to the control group (7.62 ± 1.77 × 10^3^ and 4.51 ± 1.17 × 10^3^ cells/cm^2^, respectively, *p* = 0.008; Figure 8c). In contrast, there was no significant difference in the percentage of TH^+^NURR1^+^ cells among FOXA2^+^ cells between the two groups (8.89 ± 1.89% and 7.72 ± 0.93%, respectively, *p* = 0.308; Figure 8d). These results suggested that SLITRK6 supports the survival and/or proliferation of FOXA2^+^ DA progenitors, thereby increasing the number of TH^+^NURR1^+^ DA neurons. We also assessed the length of TH^+^ fibers in both conditions (*n* = 5, each). The average fiber length was significantly longer in the SLITRK6 group compared to the control group (1.01 ± 0.45 × 10^2^ and 5.81 ± 1.86 × 10 µm, respectively, *p* = 0.024; Figure 8e).

## 4 DISCUSSION

Our results show that the administration of ZNS increased the number of survived DA neurons after the transplantation of human‐iPS cell‐derived DA progenitors into rat striatum. We found that 4% of DA progenitors were proliferating at the time of injection. One month after the transplantation, the number of mature DA neurons (TH^+^NURR1^+^) in the graft had increased by ZNS, but the number of total survived cells (HNA^+^) had not. However, the number of proliferating KI67^+^ cells slightly decreased in ZNS‐treated rats. These findings suggested that ZNS promoted the maturation of grafted DA progenitors. At 4 months, however, the number of HNA^+^ cells was higher in ZNS‐treated rats, and there was no difference in the percentage of TH^+^NURR1^+^ cells among HNA^+^ cells. These results suggest that ZNS supports the survival and/or proliferation of DA progenitors/neurons over longer periods.

Previous reports suggested two potential mechanisms for the neuroprotective effects of ZNS: direct (Condello et al., 2013; Kawajiri et al., 2010) and indirect (Asanuma et al., 2010; Choudhury et al., 2010). In this study, we did not observe direct effects on DA progenitors (Figure 4). Therefore, we focused on the effect of ZNS on the host brain, finding by a microarray analysis that ZNS increased the expression of *SLITRK6* in the rat striatum (Figure 5). SLITRK family proteins are single‐pass transmembrane proteins that resemble SLIT in the outer‐membrane portion and NTRK in the inner‐membrane portion (Tekin et al., 2013). The SLITRK family consists of six proteins, SLITRK1–6, which are reported to bind to leukocyte common antigen‐related receptor protein tyrosine phosphatases (LAR‐RPTPs), which are synaptic adhesion molecules related to synapse development. Although the binding regions of SLITRKs and LAR‐RPTP have been identified, the regions are not conserved in SLITRK6. SLITRK6 is expressed in the thalamus of mouse and zebrafish (Beaubien & Cloutier, 2009; Round, Ross, Angel, Shields, & Lom, 2014) and also in the human putamen (Aruga, Yokota, & Mikoshiba, 2003), but its target is not known (Um et al., 2014; Um & Ko, 2013). SLITRK6 is reported to facilitate synapse formation, support the survival of neural cells, and control axonal extension, although its effects on neurite extension are controversial (Aruga et al., 2003; Katayama et al., 2009; Tekin et al., 2013).

We confirmed that CHAT^+^ cholinergic interneurons express SLITRK6 in the rat striatum. Cholinergic interneurons consist of about 1% of all neurons in the striatum (Lim, Kang, & McGehee, 2014). They extend neurites to the whole striatum and form synapses with the axons of DA neurons in the substantia nigra (Round et al., 2014). They also show synchronous excitation in response to input from thalamostriatal axons, which are positive for nicotinic acetylcholine receptors, thereby controlling DA release from DA neurons in the substantia nigra (Threlfell et al., 2012). We showed that co‐culture with SLITRK6‐HEK293T cells increased the survival of DA progenitors and consequently TH^+^NURR1^+^ DA neurons. In addition, the length of TH^+^ neurites became longer on SLITRK6‐HEK293T cells. These findings suggest that SLITRK6 protein expressed on the surface of cholinergic neurons directly acts on the grafted cells.

One important limitation of this study is the small sample size, which may reduce the reproducibility and generalizability of our findings. Especially, the small sample size underpowered our *in vitro* analysis, in which we could not detect the direct effect of ZNS. Nevertheless, we confirmed the upregulation of SLITRK6 in the striatum of ZNS‐treated rats by quantitative RT‐PCR and immunohistology. Another limitation is that we only used female rats in this study. The survival rate of DA neurons significantly differs between genders when neurotoxin 6‐hydroxydopamine was administered (Murray et al., 2003). That study suggested estrogen may have a neuroprotective effect, which could explain the difference. Because we used only female animals in this study, future experiments should investigate male rats.

## 5 CONCLUSION

Zonisamide promoted the survival of DA neurons after the transplantation of human‐iPS cell‐derived DA progenitors in rat striatum. This supportive effect of ZNS is suggested to be caused by the increased expression of SLITRK6 in the host striatum. ZNS is already available in the clinic and is expected to be useful for future PD cell therapy.

## DECLARATION OF TRANSPARENCY

The authors, reviewers and editors affirm that in accordance to the policies set by the *Journal of Neuroscience Research*, this manuscript presents an accurate and transparent account of the study being reported and that all critical details describing the methods and results are present.

## CONFLICT OF INTEREST

J.T. has a financial conflict of interest concerning this study, as below. Joint research funds: Sumitomo Dainippon Pharma Co., Ltd.

## AUTHOR CONTRIBUTIONS

All authors had full access to all the data in the study and take responsibility for the integrity of the data and the accuracy of the data analysis. *Conceptualization*, Y.M. and J.T.; *Methodology*, Y.M.; *Investigation*, Y.M., B.S., and K.N.; *Formal Analysis*, Y.M.; *Resources*: Y.M., B.S., and K.N.; *Writing – Original Draft*, Y.M. and J.T.; *Writing – Review & Editing*, Y.M., T.K., and J.T.; *Visualization*, Y.M.; *Supervision*, J.T.; *Funding Acquisition*, J.T.

## Supporting information

Table S1. List of antibodies

Table S2. List of primers

Transparent Peer Review Report

Transparent Science Questionnaire for Authors

## Data Availability

The microarray data are available from the Gene Expression Omnibus (GEO database, http://www.ncbi.nlm.nih.gov/geo/) with the accession number GSE112624.
