# Supplementary material for: Zonisamide promotes survival of human‐induced pluripotent stem cell‐derived dopaminergic neurons in the striatum of female rats
Source: J Neurosci Res. 2020 Jun 7;98(8):1575–87. doi: 10.1002/jnr.24668 (PMC7497107; doi:10.1002/jnr.24668)
Supplement: Supplementary file 3 — Transparent Peer Review Report [file JNR-98-1575-s003.docx]

Zonisamide promotes survival of human induced pluripotent stem cell-derived dopaminergic neurons in the striatum of female rats

Miyawaki, Yoshifumi; Samata, Bumpei; Kikuchi, Tetsuhiro; Nishimura, Kaneyasu; Takahashi, Jun

_____________________________________________________________________________________

| Review timeline: | Submission date: 23/08/19  Editorial Decision: 17/10/19; Minor revision  Revision Received: 30/12/19  Editorial Decision: 17/02/20; Minor revision  Revision Received: 14/03/20  Editorial Decision: 05/05/20; Accept with minor edits  Revision Received: 15/05/20  Accepted: 17/05/2020 |  |
| --- | --- | --- |
|  |  |  |

Editor: Sheila Fleming

Reviewer 1: Muhammad Hossain

Reviewer 2: Lachlan Tompson

Reviewer 3: Agnete Kirkeby

_____________________________________________________________________________________

1st Editorial Decision	17/10/2019

_____________________________________________________________________________________

17-Oct-2019

Dear Dr Kikuchi:

Thank you for submitting your manuscript to the Journal of Neuroscience Research. We've now received the reviewer feedback and have appended those reviews below. Overall, the manuscript was well written, designed, and considered important to the field. There are minor suggested changes that do still need to be addressed though. Please provide a point-by-point response for each comment for all of the reviews. I expect that these points should be relatively straightforward to address. If there are any questions or points that are problematic, please feel free to contact me. I am glad to discuss.

We ask that you return your manuscript within 30 days. Please explain in your cover letter how you have changed the present version. If you require longer than 30 days to make the revisions, please contact Dr. Eric Prager ([eprager@wiley.com](mailto:eprager@wiley.com)). You can submit your revised manuscript directly by clicking on the following link: *** PLEASE NOTE: This is a two-step process. After clicking on the link, you will be directed to a webpage to confirm. *** 

<https://mc.manuscriptcentral.com/jnr?URL_MASK=f10c447823b7489d92a8f567fddfcb98>

Thank you again for your submission to the Journal of Neuroscience Research; we look forward to reading your revised manuscript.

Best Wishes,

Dr Sheila Fleming
Associate Editor, Journal of Neuroscience Research

Dr Eric Prager
Editor-in-Chief, Journal of Neuroscience Research

Additional Editorial Comments:
1) The authors report using female F344-Il2rg x SCID rats for transplantation, but do not report why only females. The authors also do not report ethical approval and will need to do so. The microarray experiments used a different strain of the f344 rats, but again the authors do not state why. 

2) In many of the experiments, n of 4 or 2 were used. Please justify this sample size as it is low and we are concerned the findings are underpowered. 

3) Authors need to report their procedures for cell counts, replicates, etc. All procedures must be described in full detail.

Reviewer: 1

Comments to the Author
This manuscript investigates the effects of zonisamide (ZNS) on survival of iPS cell-derived human dopaminergic neurons transplanted into rat striatum. In this study, authors show that ZNS promoted the survival of these DA progenitors by increased the expression of SLITRK6. However, the article is well written, there are some specific comments that need to be addressed.

1.	Do these iPS cell-derived human dopaminergic progenitor cells express any sodium or calcium channels? Since zonisamide exerts it effects by blocking of these channels, there might be a co-relation between sodium or calcium channels expression and up regulation of SLITRK6 expression on the survived iPS cell-derived human DA progenitor cell. This piece of information is very important and may provide an important insight into the mechanism by which ZNS upregulates SLITRK6. 

2.	In page 9, the authors mentioned 5×105 cells were injected into the striatum, but they did not mention how much volume was injected. Are the cells were suspended in 0.9% NaCl or 1X PBS? Please clarify, what saline solution was used and how many µL was injected for 5×105 cells. Author should also provide the rationale for why they transplanted into the striatum but not into the substantia nigra.  

3.	It is not clear whether “n = 3” represents the number experiments or the number wells from one experiment. Authors should describe exactly how many times each experiment was repeated and how many replications (wells) were used per experiment. Data for TH + cells is missing in the figure 1C. Please revise this figure with data for TH + cell counts. Furthermore, for better representation, authors should represent the data from KI67 and FOXA2 in a bar graph as well. Please revise this figure accordingly. 

4.	It seems that the animals were treated with 30 to 60 mg/kg of zonisamide for 1-4 months after transplantation of iPS cell-derived human dopaminergic progenitor cells. But no data were found for the survival of injected DA progenitors with 30 mg/kg of zonisamide at 4-month time point (Figure 2). The author should revise this figure additional data from at 4-month time point, otherwise, please explain why these data were not added.


Reviewer: 2

Comments to the Author
This is a well presented study on the influence of systemically administered ZNS on the survival of DA neurons generated from iPS cells after intracerebral grafting. The methods are appropriate, the data is novel and generally well presented and the conclusions are mostly supported by the data. Overall it is in reasonably shape for publication in JNR although there are some points that need addressing:

FoxA2+ cells are referred to a number of times as DA progenitors. This is not strictly correct. FoxA2 merely implies a ventral identity and does not identify DA phenotype. This should be reworded throughout and/or the use of FoxA2 given more justification.

For the same reasons, the use of FoxA2 and Ki67 is not a measure of proliferating DA progenitors and this should be made clear. It would be useful to also represent Ki67 cells as a fraction of HNA cells in order to contextualise the FoxA2 results. Furthermore, this readout is at best only an indirect measure of any potential proliferative impact of ZNS. It does not account for the possibility that ZNS has an early proliferative impact that is no longer detectable when the animals are perfused for histology. A more accurate assessment would require birthdating (e.g. BrdU/EdU) during the period of ZNS administration.

Labelling of the 1month and 4month data within the figures would be useful.

The quality of the panels in Figure 3a appears dramatically lower than for the same data in 3c.   

Reviewer: 3

Comments to the Author
The study by Miyawaki et al. systematically analyses the effect of Zonisamide on graft survival and DA yield of hPS-derived cells after transplantation to the rodent striatum. The study is well performed and it addresses a highly relevant and important question in the field of cell therapy for PD, namely that there is a significant loss of cells upon transplantation. Therefore, is great interest in finding adjunct treatments which can enhance the survival and DA maturation of stem cell grafts. Indeed, the use of Zonisamide as proposed here is highly relevant for this purpose, as it is a licensed drug which is approved for use in PD patients, so it would be straightforward to implement in clinical trials of stem cell transplants. The manuscript deserves publication, and I raise only a few points for improvement.
The manuscript finds that Zonisamide treatment increases the yield of TH+ cells in the grafts and also increases graft size at 4 months. The reason for this is unclear, but microarray data and in vitro analyses suggests that this is due to an upregulation of Slitrk6 on striatal interneurons, which in turn causes increased survival and/or proliferation selectively of FOXA2+ progenitors. Since there is currently a gap and some discrepancy in the conclusions between the in vitro and in vivo data, it should be assessed if the results from the in vitro data are also recapiutaled in vivo. For this, the grafts at 1M and 4Mshould be assessed for the total number of FOXA2+ cells and the number of FOXA2+ cells out of total HNA+ cells. Although it is already shown that there is not increase in FOXA2+/KI67+ cells in the grafts after Zonisamide treatment, an analysis of total FOXA2+ cells would answer whether Zonisamide also in vivo increases the survival selectively of FOXA2+ progenitors and whether this is a possible explanation to the increase in TH+/NURR1+ cells found in the grafts, as is hypothesized from the in vitro studies. In addition, some minor points:
-	Page 6, line 94: It is stated that Zonisamide is “used to treat PD patients” A more correct statement here would be that it is used as a “symptomatic treatment” for PD to clarify that it is not used as a disease-modifying treatment. It may be relevant to cite a review on Zonisamide in PD here.
-	Abstract + significance statement + p5, line 87: It is stated several times that only 10% of cells survive after transplantation to the brain, however, there are several problems/ambiguities to these statements and the referenced studies. On page 4 and 5 it is stated that the “number of survived PSC-derived cells after transplantation is approximately 10% of the total number of grafted cells”, however, in the abstract this statement is specified as only 10%  transplanted cells are “survived DA neurons”. There is a significant difference between these two statements – do the authors mean that only 10% of all transplanted cells survive, or that the grafts give rise to DA neurons in a quantity which is 10% of total number of grafted cells? To my knowledge, there is no study which has quantified cells survival after transplantation – such an analysis would require quantitative assessment of the grafted cells 1-2 days after transplantation to assess direct cell survival. Any analysis performed at later timepoints will assess the number of cells in the grafts which is a combined effect of cell death and cell proliferation – this can be more correctly termed as “yield” after transplantation.
-	Furthermore, the referenced studies on p5 do not support the statement on p5 (“number of survived PSC-derived cells after transplantation is approximately 10% of the total number of grafted cells”, ref Doi, Kikuchi, Hagell) as none of the referenced studies have assessed direct cell survival after transplantation:
o	The Doi et al study assessed TH+ yield in the grafts to be between 0.3-1.8% of the total number of implanted cells (i.e. between 1000-7000 TH+ neurons in the grafts from 400.000 grafted cells). The total number of surviving cells was not assessed. 
o	The Hagell/Brundin study assessed fetal-derived grafts and not PSC-derived grafts, and the percentage of surviving cells from total number of cells is not known from these studies. 
o	The Kikuchi et al study assessed TH+ yield in the primate grafts to be around 2% of the total number of implanted cells (i.e. avg around 100.000 TH+ neurons in the grafts from 2.4M grafted cells). The total number of surviving cells was not assessed. 
-	P17/18: It is concluded from the in vitro studies that “SLITRK6 supports the survival of FOXA2+ DA progenitors”. However, the results do not allow to conclude whether the effect of SLITRK6 is to increase survival or proliferation of the FOXA2+ progenitors. A more correct statement would be  “SLITRK6 supports the survival and/or proliferation of FOXA2+ DA progenitors”. The same comment is true for the discussion, p18, line 375-376
-	The reference bibliography is not set up correctly. References are cited by name in the text, but the reference list is ordered by numbering.

____________________________________________________________________________________

Authors’ Response	30/12/2019

_____________________________________________________________________________________

Additional Editorial Comments:
1) The authors report using female F344-Il2rg x SCID rats for transplantation, but do not report why only females. The authors also do not report ethical approval and will need to do so. The microarray experiments used a different strain of the f344 rats, but again the authors do not state why. 

It is well known that the neurodegenerative effect of 6-hydroxy dopamine differs between male and female animals. Furthermore, the size of the brain also differs between both sexes. We selected female animals in this study because female rats have been used in many studies to assess PD symptoms (Brundin et al, 1986; Samata et al, 2015). Accordingly, we added the following statement in Materials and Method in the revised manuscript:

In this study, female rats were used because female rats are suitable for behavioral analysis after transplantation of iPSC derived cells (Brundin et al, 1986, Samata et al, 2015).

As for the ethical approval, we state that "This study was approved by the ethics committee of Kyoto University, Kyoto, Japan" in the first section of Materials and Methods in the manuscript. We also added the following sentence in the "Cell transplantation" section of Materials and Methods:

All animals were cared for and handled according to the Guidelines for Animal Experiments of Kyoto University.

2) In many of the experiments, n of 4 or 2 were used. Please justify this sample size as it is low and we are concerned the findings are underpowered. 

Thank you for the comment. Although the n for any one experiment is not sufficient to lead to strong conclusions, we evaluated one phenomenon from multiple aspects, such as immunostaining, microarray, and qPCR. Moreover, the most crucial part of the manuscript, in vivo study, was performed with more animals (n = 5 to 8).

3) Authors need to report their procedures for cell counts, replicates, etc. All procedures must be described in full detail.

We apologize for the confusion. We added the following statements to Material and Methods in the revised manuscript:

Counting the number of TH+, NURR1+, FOXA2+, and Ki67+ cells was performed using 30 µm sections of the brain. One slice out of eight continuous sections was used for the cell count, and at least two sections per animal was counted. For human nuclei and SLITRK6, at least 300 cells in randomly selected 20× fields were counted.

Reviewer: 1

Comments to the Author
This manuscript investigates the effects of zonisamide (ZNS) on survival of iPS cell-derived human dopaminergic neurons transplanted into rat striatum. In this study, authors show that ZNS promoted the survival of these DA progenitors by increased the expression of SLITRK6. However, the article is well written, there are some specific comments that need to be addressed.

1.	Do these iPS cell-derived human dopaminergic progenitor cells express any sodium or calcium channels? Since zonisamide exerts it effects by blocking of these channels, there might be a co-relation between sodium or calcium channels expression and up regulation of SLITRK6 expression on the survived iPS cell-derived human DA progenitor cell. This piece of information is very important and may provide an important insight into the mechanism by which ZNS upregulates SLITRK6. 

Although we did not assess sodium and calcium channels in the grafts, we performed a microarray analysis of iPSC-derived DA progenitor cells *in vitro*. In this study, day 28 spheres were administered 0, 0.05, 0.5, and 5 µM ZNS for 11 days, and the samples were analyzed (n=2). The following graphs show the expression of T type calcium channels and voltage-dependent sodium channels. There was no specific tendency of these channels being expressed after the administration of ZNS.

2.	In page 9, the authors mentioned 5×105 cells were injected into the striatum, but they did not mention how much volume was injected. Are the cells were suspended in 0.9% NaCl or 1X PBS? Please clarify, what saline solution was used and how many µL was injected for 5×105 cells. Author should also provide the rationale for why they transplanted into the striatum but not into the substantia nigra.  

We apologize for the confusion. 5×10^5^ cells / 2µL PBS were injected. We injected the cells into the striatum because donor cells were injected into the striatum, which is a target of dopaminergic neurons in the substantia nigra, in previous fetal cell transplantations for Parkinson's disease. We revised the following statements in Materials and Methods in the revised manuscript accordingly:

Original: The neurospheres (5.0×10^5^ cells per animal) were injected...

Revised: The neurospheres (5.0×10^5^ cells in 2 µL PBS solution per animal) were injected...

3.	It is not clear whether “n = 3” represents the number experiments or the number wells from one experiment. Authors should describe exactly how many times each experiment was repeated and how many replications (wells) were used per experiment. Data for TH + cells is missing in the figure 1C. Please revise this figure with data for TH + cell counts. Furthermore, for better representation, authors should represent the data from KI67 and FOXA2 in a bar graph as well. Please revise this figure accordingly. 

We apologize for the confusion. “n = 3” represents the number of independent experiments. At least 300 cells from one well per experiment were assessed. We added the following statement in Material and Methods and revised Figure 1 in the revised manuscript according to the reviewer's comments:

n represents the number of independent experiments.

4.	It seems that the animals were treated with 30 to 60 mg/kg of zonisamide for 1-4 months after transplantation of iPS cell-derived human dopaminergic progenitor cells. But no data were found for the survival of injected DA progenitors with 30 mg/kg of zonisamide at 4-month time point (Figure 2). The author should revise this figure additional data from at 4-month time point, otherwise, please explain why these data were not added.

As shown in figure 2d, more TH+NURR1+ cells survived in the ZNS groups than the vehicle group, and there was no significant difference between the ZNS 30 and ZNS 60 groups. Based on these results, we decided to perform 4 months observation only in the ZNS 60 group. We revised the following statement in "ZNS promoted survival of DA neurons four months after transplantation" section in Results in the revised manuscript accordingly:

Original: Next, we examined the survival of...

Revised: From the above results, we continued observation of the ZNS 60 mg/kg group. We examined the survival of...

Reviewer: 2

Comments to the Author
This is a well presented study on the influence of systemically administered ZNS on the survival of DA neurons generated from iPS cells after intracerebral grafting. The methods are appropriate, the data is novel and generally well presented and the conclusions are mostly supported by the data. Overall it is in reasonably shape for publication in JNR although there are some points that need addressing:

FoxA2+ cells are referred to a number of times as DA progenitors. This is not strictly correct. FoxA2 merely implies a ventral identity and does not identify DA phenotype. This should be reworded throughout and/or the use of FoxA2 given more justification.

We agree with the reviewer that FoxA2 is a mere marker for ventral identity. However, in our method with Corin sorting, most FoxA2+ cells are also Lmx1a+ cells (Doi et al. 2014), and we define this population as DA progenitors. Therefore, in our case, "FoxA2+ DA progenitor" does not mean merely FoxA2+ cells, but Corin-sorted, dopamine-oriented FoxA2+ cells. We added the following statement in "Differentiation of human iPS cells to DA progenitors" in Results in the revised manuscript:

With this method, most of the differentiated cells were LIM homeobox transcription factor 1 alpha (LMX1A, midbrain marker)+ and FOXA2+ DA neuronal progenitors (Doi et al, 2014).

For the same reasons, the use of FoxA2 and Ki67 is not a measure of proliferating DA progenitors and this should be made clear. It would be useful to also represent Ki67 cells as a fraction of HNA cells in order to contextualise the FoxA2 results. Furthermore, this readout is at best only an indirect measure of any potential proliferative impact of ZNS. It does not account for the possibility that ZNS has an early proliferative impact that is no longer detectable when the animals are perfused for histology. A more accurate assessment would require birthdating (e.g. BrdU/EdU) during the period of ZNS administration.

Thank you for the helpful comment. We added quantification of the FOXA2/HNA cells in Figure 3. With regard to the importance of the BrdU/EdU experiments, we agree with the reviewer. However, BrdU has mutagenicity and is potentially harmful for DA progenitors. Therefore, we think it is a great challenge to administer BrdU and ZNS together.

Labelling of the 1month and 4month data within the figures would be useful.

We revised Figures 2 and 3 accordingly.

The quality of the panels in Figure 3a appears dramatically lower than for the same data in 3c.

We revised figure 3a accordingly.

Reviewer: 3

Comments to the Author
The study by Miyawaki et al. systematically analyses the effect of Zonisamide on graft survival and DA yield of hPS-derived cells after transplantation to the rodent striatum. The study is well performed and it addresses a highly relevant and important question in the field of cell therapy for PD, namely that there is a significant loss of cells upon transplantation. Therefore, is great interest in finding adjunct treatments which can enhance the survival and DA maturation of stem cell grafts. Indeed, the use of Zonisamide as proposed here is highly relevant for this purpose, as it is a licensed drug which is approved for use in PD patients, so it would be straightforward to implement in clinical trials of stem cell transplants. The manuscript deserves publication, and I raise only a few points for improvement.
The manuscript finds that Zonisamide treatment increases the yield of TH+ cells in the grafts and also increases graft size at 4 months. The reason for this is unclear, but microarray data and in vitro analyses suggests that this is due to an upregulation of Slitrk6 on striatal interneurons, which in turn causes increased survival and/or proliferation selectively of FOXA2+ progenitors. Since there is currently a gap and some discrepancy in the conclusions between the in vitro and in vivo data, it should be assessed if the results from the in vitro data are also recapiutaled in vivo. For this, the grafts at 1M and 4Mshould be assessed for the total number of FOXA2+ cells and the number of FOXA2+ cells out of total HNA+ cells. Although it is already shown that there is not increase in FOXA2+/KI67+ cells in the grafts after Zonisamide treatment, an analysis of total FOXA2+ cells would answer whether Zonisamide also in vivo increases the survival selectively of FOXA2+ progenitors and whether this is a possible explanation to the increase in TH+/NURR1+ cells found in the grafts, as is hypothesized from the in vitro studies.

Thank you for your constructive comments. Essentially, with our method, more than 90% of cells were FOXA2+. We added the FOXA2+/HNA+ cell count in Figure 2k and added the following statement in Results:

Additionally, most of the HNA+ cells were FOXA2+ (91.3 ± 1.1% and 90.1 ± 2.3% for vehicle and ZNS 60 mg/kg, respectively, p = 0.62; Figure 2k).

In addition, some minor points:
-	Page 6, line 94: It is stated that Zonisamide is “used to treat PD patients” A more correct statement here would be that it is used as a “symptomatic treatment” for PD to clarify that it is not used as a disease-modifying treatment. It may be relevant to cite a review on Zonisamide in PD here.

We revised the sentence as follows:

Original: Zonisamide ... is used to treat PD patients ...

Revised: Zonisamide ... is used as a symptomatic treatment for PD patients ...

-	Abstract + significance statement + p5, line 87: It is stated several times that only 10% of cells survive after transplantation to the brain, however, there are several problems/ambiguities to these statements and the referenced studies. On page 4 and 5 it is stated that the “number of survived PSC-derived cells after transplantation is approximately 10% of the total number of grafted cells”, however, in the abstract this statement is specified as only 10%  transplanted cells are “survived DA neurons”. There is a significant difference between these two statements – do the authors mean that only 10% of all transplanted cells survive, or that the grafts give rise to DA neurons in a quantity which is 10% of total number of grafted cells? To my knowledge, there is no study which has quantified cells survival after transplantation – such an analysis would require quantitative assessment of the grafted cells 1-2 days after transplantation to assess direct cell survival. Any analysis performed at later timepoints will assess the number of cells in the grafts which is a combined effect of cell death and cell proliferation – this can be more correctly termed as “yield” after transplantation.

Thank you for your comments.

We revised the relevant statements in the Abstract, Significant Statement, and Introduction as follows:

Original: However, the number of survived DA neurons is only approximately 10% of the number of transplanted cells.

Revised: However, the survival of DA progenitors is low, and the final yield of dopaminergic neurons is only approximately 0.3–2% the number of transplanted cells.

Original: Only about 10% of transplanted cells, however, survive in the transplanted animal brains. This low survivability of the graft demands...

Revised: The survival of transplanted cells, however, is low, and the final yield of dopaminergic neurons is only about 0.3–2% of the transplanted cells. This low yield demands...

Original: Another concern is that the number of survived PSC-derived cells after transplantation is approximately 10% of the total number of grafted cells (Doi et al., 2014; Kikuchi et al., 2017; Hagell & Brundin, 2001)

Revised: Another concern is that only a small fraction of grafted cells survive after transplantation, and the final yield of dopaminergic neuron is only about 0.3–2% of the transplanted cells (Doi et al., 2014; Kikuchi et al., 2017).

-	Furthermore, the referenced studies on p5 do not support the statement on p5 (“number of survived PSC-derived cells after transplantation is approximately 10% of the total number of grafted cells”, ref Doi, Kikuchi, Hagell) as none of the referenced studies have assessed direct cell survival after transplantation:
o	The Doi et al study assessed TH+ yield in the grafts to be between 0.3-1.8% of the total number of implanted cells (i.e. between 1000-7000 TH+ neurons in the grafts from 400.000 grafted cells). The total number of surviving cells was not assessed. 
o	The Hagell/Brundin study assessed fetal-derived grafts and not PSC-derived grafts, and the percentage of surviving cells from total number of cells is not known from these studies. 
o	The Kikuchi et al study assessed TH+ yield in the primate grafts to be around 2% of the total number of implanted cells (i.e. avg around 100.000 TH+ neurons in the grafts from 2.4M grafted cells). The total number of surviving cells was not assessed.

We removed the Hagell/Brundin study from references in this context.

-	P17/18: It is concluded from the in vitro studies that “SLITRK6 supports the survival of FOXA2+ DA progenitors”. However, the results do not allow to conclude whether the effect of SLITRK6 is to increase survival or proliferation of the FOXA2+ progenitors. A more correct statement would be “SLITRK6 supports the survival and/or proliferation of FOXA2+ DA progenitors”. The same comment is true for the discussion, p18, line 375-376

We revised the sentences as follows:

Original: These results suggested that SLITRK6 supports the survival of FOXA2+ DA progenitors...

Revised: These results suggested that SLITRK6 supports the survival and/or proliferation of FOXA2+ DA progenitors...

Original: These results suggest that ZNS supports the survival of DA progenitors/neurons over longer periods.

Revised: These results suggest that ZNS supports the survival and/or proliferation of DA progenitors/neurons over longer periods.

-	The reference bibliography is not set up correctly. References are cited by name in the text, but the reference list is ordered by numbering.

We revised the References accordingly.

_____________________________________________________________________________________

2^nd^ Editorial Decision	18/02/2020

_____________________________________________________________________________________

18-Feb-2020

Dear Dr Kikuchi:

Thank you for submitting your manuscript to the Journal of Neuroscience Research. We've now received the reviewer feedback and have appended those reviews below. 

Although some improvement in the quality of paper is noted from the previous version, The manuscript still requires substantial editing and proofreading. Please consider reviewer 2's additional minor comments and address them in a point by point manner.

I expect that these points should be relatively straightforward to address. If there are any questions or points that are problematic, please feel free to contact me. I am glad to discuss. .

We ask that you return your manuscript within 30 days. Please explain in your cover letter how you have changed the present version. If you require longer than 30 days to make the revisions, please contact Dr Cristina Ghiani ([cghiani@mednet.ucla.edu](mailto:cghiani@mednet.ucla.edu)). You can submit your revised manuscript directly by clicking on the following link: *** PLEASE NOTE: This is a two-step process. After clicking on the link, you will be directed to a webpage to confirm. *** 

<https://mc.manuscriptcentral.com/jnr?URL_MASK=66bdabcc5a8742339ac3c2d28362aeb3>

Thank you again for your submission to the Journal of Neuroscience Research; we look forward to reading your revised manuscript.

Best Wishes,

Dr Sheila Fleming
Associate Editor, Journal of Neuroscience Research

Dr Cristina Ghiani
Co-Editor-in-Chief, Journal of Neuroscience Research


Associate Editor: Fleming, Sheila
Comments to the Author:
The revised manuscript is greatly improved but please see reviewer 2's additional minor comments and address them in a point by point manner.


Statistics Editor: McArthur, David
Comments to the Author:

With quite small sample sizes, whatever findings are reported must be interpreted with caution as reproducibility and generalizabilty may be very limited  Kindly discuss this limitation in the narrative, not merely in the responses to reviewer.  For example, the claim that at least 300 cells were assessed is critical to your mission.  Why Figure 4C shows 4 datapoints per segment but the caption indicates "n=3" is not clear.

The use of SEM is not provided with a rationale.  SD's are preferred.  SEMS's calculated on separate data distributions that all have identical means and identical variances will be the same only if the number of cases in all groups is also the same.  Thus SEMs cannot be interpreted as a stable reflection of variability for a given set of data distributions, and the visualizations using SEMs are thus also not stable.  It is for these reasons that 21st century statistical thinking has pushed for SDs.

Each p-value in the Results narrative must be accompanied by the appropriate statistical results:  For anova we would expect "F(df1,df2) = x.xx, p = 0.yyy" for example, e.g., standard notation. Kindly supply these findings everywhere such test results are appropriate.  The figure captions use the asterisk version of p-values, which is not a substitute for presenting the findings completely.  The statistics cannot be checked without this.

This journal requests that p-values be shown using a consistent decimal exactness: numerous values in this text are given to varying number of digits or they are expressed only as "P<0.05" and the like.  Kindly choose a decimal, say 3, and stay with it throughout, with "p<0.001" reserved as appropriate.

Reviewer: 1

Comments to the Author
All concerns addressed.

Reviewer: 2

Comments to the Author
The revised version of the manuscript adequately addresses the issues raised by reviewer. Although some improvement in the quality of paper is noted from previous version, the paper still suffers from poor writing throughout the manuscript. 

1.	The manuscript requires substantial editing and proofreading to improve errors of syntax throughout the manuscript.
2.	Lines 48-50: Reference is required for this statement. Lines 50-51: “It is not known, however, whether ZNS exerts the same effect on human DA neurons” is vague, please delete this sentence.
3.	Please use citation correctly. There are numerous errors with citations (i.e. Piotr Czapin´ski, Barbara Blaszczyk, & Stanislaw J. Czuczwar, 2005)” throughout the manuscript.
4.	In method section, please include how many total animals were used in this study. Mention the unit of penicillin/streptomycin used. Please include Cat #, source, and place for major instruments, kits, and antibodies you used in this study. Please mention incubation temperature for cell sorting (Lines 154) and immunostaining (Line 189). Replace the word samples with cells (Lines 195, 198). Line 186: sentence is incomplete, will be -----mounted on slides. Line 236-: the sentence should be read as “Three days prior to co-culture, ------.” Line 246: It should be “After immunostaining, TH+ fibers--------.” 
5.	The result section is too long. There is numerous repetition from methods (i.e. Lines 292-294). Please eliminate information that has already been stated in methods. Line 378: 102 will be 10 μm.
6.	Table 1. (antibodies) and Table 2 (primers) should go to supplemental file.   
7.	Figure 8 must me be revised as the images of top panels are very poor (over exposed) 
8.	Discussion needs to appropriately revised. Lines 387-388: Author can say “However, number of proliferating KI67+ cells slightly decreased in ZNS-treated rats.


Reviewer: 3

Comments to the Author
The authors have adequately addressed the reviewers' concerns and comments.

_____________________________________________________________________________________

Authors’ Response	14/03/2020

_____________________________________________________________________________________

Statistics Editor: McArthur, David
Comments to the Author:

With quite small sample sizes, whatever findings are reported must be interpreted with caution as reproducibility and generalizabilty may be very limited. Kindly discuss this limitation in the narrative, not merely in the responses to reviewer.  For example, the claim that at least 300 cells were assessed is critical to your mission.  Why Figure 4C shows 4 datapoints per segment but the caption indicates "n=3" is not clear.

Thank you for the suggestion. We have added a new paragraph about this limitation at the end of the Discussion.

Regarding Figure 4C, the caption was wrong. The caption has been corrected to read "n = 4".

The use of SEM is not provided with a rationale.  SD's are preferred.  SEMS's calculated on separate data distributions that all have identical means and identical variances will be the same only if the number of cases in all groups is also the same.  Thus SEMs cannot be interpreted as a stable reflection of variability for a given set of data distributions, and the visualizations using SEMs are thus also not stable.  It is for these reasons that 21st century statistical thinking has pushed for SDs.

As suggested by the reviewer, we use SD in the revised manuscript.

Each p-value in the Results narrative must be accompanied by the appropriate statistical results:  For anova we would expect "F(df1,df2) = x.xx, p = 0.yyy" for example, e.g., standard notation. Kindly supply these findings everywhere such test results are appropriate.  The figure captions use the asterisk version of p-values, which is not a substitute for presenting the findings completely.  The statistics cannot be checked without this.

As suggested by the reviewer, we have added the appropriate statistical results throughout the revised manuscript.

This journal requests that p-values be shown using a consistent decimal exactness: numerous values in this text are given to varying number of digits or they are expressed only as "P<0.05" and the like.  Kindly choose a decimal, say 3, and stay with it throughout, with "p<0.001" reserved as appropriate.

As suggested by the reviewer, we use 3 decimals in the revised manuscript.

Reviewer: 2

Comments to the Author
The revised version of the manuscript adequately addresses the issues raised by reviewer. Although some improvement in the quality of paper is noted from previous version, the paper still suffers from poor writing throughout the manuscript. 

1.	The manuscript requires substantial editing and proofreading to improve errors of syntax throughout the manuscript.

We have thoroughly reviewed the manuscript for syntax errors.

2.	Lines 48-50: Reference is required for this statement. Lines 50-51: “It is not known, however, whether ZNS exerts the same effect on human DA neurons” is vague, please delete this sentence.

As suggested by the reviewer, we deleted the sentence.

3.	Please use citation correctly. There are numerous errors with citations (i.e. Piotr Czapin´ski, Barbara Blaszczyk, & Stanislaw J. Czuczwar, 2005)” throughout the manuscript.

We apologize for the inconsistent citations. We have corrected them in the revised manuscript.

4.	In method section, please include how many total animals were used in this study. Mention the unit of penicillin/streptomycin used. Please include Cat #, source, and place for major instruments, kits, and antibodies you used in this study. Please mention incubation temperature for cell sorting (Lines 154) and immunostaining (Line 189). Replace the word samples with cells (Lines 195, 198). Line 186: sentence is incomplete, will be -----mounted on slides. Line 236-: the sentence should be read as “Three days prior to co-culture, ------.” Line 246: It should be “After immunostaining, TH+ fibers--------.”

We revised the manuscript according to the reviewer's comments.

5.	The result section is too long. There is numerous repetition from methods (i.e. Lines 292-294). Please eliminate information that has already been stated in methods. Line 378: 102 will be 10 μm.

We have removed several statements in the Results to avoid repetition. Regarding Line 378, 102 should have read 10^2^. We apologize for the confusion.

6.	Table 1. (antibodies) and Table 2 (primers) should go to supplemental file.

We moved the tables to the supplemental file as suggested.

7.	Figure 8 must me be revised as the images of top panels are very poor (over exposed)

We revised Figure 8. We also revised Figure 4a, because it too was over exposed.

8.	Discussion needs to appropriately revised. Lines 387-388: Author can say “However, number of proliferating KI67+ cells slightly decreased in ZNS-treated rats.

We revised the manuscript according to the reviewer's comments.

_____________________________________________________________________________________

3^rd^ Editorial Decision	05/05/2020

_____________________________________________________________________________________

05-May-2020

Dear Dr Kikuchi:

Thank you for submitting your manuscript to the Journal of Neuroscience Research. We've now received the reviewer feedback and have appended those reviews below. The reviewers are overall very enthusiastic and supportive of the study, and your manuscript has been accepted pending minor modifications.

In particular, before your manuscript can be fully accepted for publication, a rationale/justification for using only females in the experiments and the limitation associated with only including one sex should be discussed.

The statement in the methods does not appropriately explain the reasoning behind your choice:" In this study, female rats were used because female rats are suitable for behavioral analysis after cell transplantation (Brundin et al, 1996; Samata et al, 2015)."

We expect that this point should be relatively straightforward to address. If there are any questions or points that are problematic, please feel free to contact me. I am glad to discuss.

We ask that you return your manuscript within 15 days. Please explain in your cover letter how you have changed the present version. If you require longer than 15 days to make the revisions, please contact Dr Cristina Ghiani (cghiani@mednet.ucla.edu).You can submit your revised manuscript directly by clicking on the following link: *** PLEASE NOTE: This is a two-step process. After clicking on the link, you will be directed to a webpage to confirm. ***

https://mc.manuscriptcentral.com/jnr?URL_MASK=3a1631a333e546c5a4519e117ab00f2b

Thank you again for your submission to the Journal of Neuroscience Research; we look forward to reading your revised manuscript.

Best Wishes,

Dr Sheila Fleming

Associate Editor, Journal of Neuroscience Research

Dr Cristina Ghiani

Editor-in-Chief, Journal of Neuroscience Research

Associate Editor: Fleming, Sheila

Comments to the Author:

Please include a rationale/justification for using only females in the experiments and the limits associated with only including one sex.

_____________________________________________________________________________________

Authors’ Response	15/05/2020

_____________________________________________________________________________________

Associate Editor: Fleming, Sheila

Comments to the Author:

Please include a rationale/justification for using only females in the experiments and the limits associated with only including one sex.

Thank you for the comment. We revised the statement in Method as follows: "In this study, we used female animals because we previously showed that xenograft transplants did not cause an immune response in female X-SCID rats (Samata et al., 2015)."

Besides, we added following statements in Discussion: "Another limitation is that we only used female rats in this study. The survival rate of dopaminergic neurons significantly differs between genders when neurotoxin 6‐hydroxydopamine was administered (Murray et al., 2003). That study suggested estrogen may have a neuroprotective effect, which could explain the difference. Because we used only female animals in this study, future experiments should investigate male rats."
